# Cryptotanshinone affects HFL-1 cells proliferation by inhibiting cytokines secretion in RAW264.7 cells and ameliorates inflammation and fibrosis in newborn rats with hyperoxia induced lung injury

**DOI:** 10.3389/fphar.2023.1192370

**Published:** 2023-07-25

**Authors:** Mengmeng Ma, Tianping Bao, Jingyan Li, Linxia Cao, Bingrui Yu, Jingjing Hu, Huaiping Cheng, Zhaofang Tian

**Affiliations:** Department of Neonatology, The Affiliated Huaian No. 1 People’s Hospital of Nanjing Medical University, Huai’an, Jiangsu, China

**Keywords:** hyperoxia, bronchopulmonary dysplasia, inflammation, fibrosis, macrophage

## Abstract

**Objective:** Bronchopulmonary dysplasia (BPD) is a common complication of prematurity and has no specific treatment option. Moreover, inflammation and fibrosis play a vital role in the development of BPD. Thus, this study aimed to explore the role of the anti-inflammatory and anti-fibrotic drug cryptotanshinone (CTS) in the treatment of inflammation and fibrosis in BPD.

**Methods:** *In vivo*, Sprague–Dawley rats (male) were divided into air, hyperoxia and CTS groups with different dose interventions (7.5, 15, and 30 mg/kg). A BPD rat model was induced by continuous inhalation of hyperoxia (95%) for 7 days, during which different doses of CTS were injected intraperitoneally. Furthermore, histological examination, hydroxyproline content measurement, Western blot and real-time quantitative polymerase chain reaction were used to detect the levels of inflammation and fibrosis in the tissues. RAW264.7 cells exposed to 95% oxygen were collected and co-cultured with fibroblasts to determine the expression levels of α-SMA, collagen-Ⅰ and MMPs. The levels of pro-inflammatory cytokines such as TNF-α, IL-6 and pro-fibrotic factor TGF-β1 in the supernatants were measured using enzyme-linked immunosorbent assay.

**Results:** Haematoxylin and eosin staining revealed that CTS reduced the inflammatory response in rat lungs. Masson staining revealed that CTS alleviated the level of pulmonary fibrosis. CTS also reduced the levels of TNF-α, IL-6 and TGF-β1 along with the expression of the fibrosis marker α-SMA in lung tissue. Similarly, *in vitro* analysis revealed that CTS decreased the levels of TNF-α, IL-6 and TGF-β1 expressed in RAW 264.7 cells, and reduced α-SMA, collagen-Ⅰ, MMPs concentrations in HFL-1 cells co-cultured with the supernatant of RAW264.7 cells after hyperoxia.

**Conclusion:** CTS can attenuate the hyperoxia-induced inflammatory response and the level of fibrosis by regulating the levels of inflammatory factors and fibrotic factor TGF-β1 expressed by macrophages, thereby highlighting the therapeutic potential of CTS in the treatment of BPD.

## 1 Introduction

Bronchopulmonary dysplasia (BPD) is one of the most common chronic diseases of prematurity. Moreover, it is a serious pulmonary complication caused by multiple factors (Owen et al., 2017), mainly alveolar destruction, fibrosis, inflammatory cell accumulation and abnormal microvascular development (Thébaud et al., 2019). BPD is a multi-response process involving inflammation and fibrosis, where inflammatory injury leads to the recruitment of macrophages and their secreted factors. The pro-inflammatory environment further induces various pathological features, such as epithelial necrosis, fibrosis and dysregulated microvascular growth (Cannavò et al., 2021; Htun et al., 2021). Meanwhile, fibrosis is a major pathological change occurring in BPD. Under hyperoxic conditions, fibroblasts affect alveolar development, repair and regeneration, thereby promoting extracellular matrix (ECM) remodelling and fibrosis development (McGowan, 2017), and such a condition further worsens the fibrotic process.

However, the pathogenesis of BPD remains unclear. Additionally, the role of different immune cells in the pathogenesis of BPD is also unclear. Recent studies have demonstrated that resident and non-resident macrophages are the main components of the inflammatory microenvironment following lung injury response (Hirani et al., 2022). In an inflammatory environment, monocytes can be recruited to the lung for conversion into alveolar macrophages (Aegerter et al., 2022). Moreover, alveolar macrophages can be activated in response to hyperoxia, leading to abnormal lung development in immature mice (Kalymbetova et al., 2018). Macrophages not only secrete pro-inflammatory cytokines such as interleukin (IL)-6, tumour necrosis factor (TNF)-α and the pro-fibrotic growth factor transforming growth factor (TGF)-β1 (Holzfurtner et al., 2022; Ozdemir et al., 2022), which regulate the intracellular immune response, but also play an essential role in the late repair phase of lung injury. These macrophage-secreted cytokines trigger fibroblasts to differentiate into activated myofibroblasts, thus promoting fibroblast recruitment and proliferation and leading to excessive collagen fiber accumulation, which eventually leads to pulmonary fibrosis (Sayan and Mossman, 2016; Barnes et al., 2019). Therefore, the regulation of macrophage function may be a potential therapeutic target for hyperoxia-induced lung injury. Currently, there is neither a definitive treatment strategy nor a preventive strategy for the treatment of BPD. Thus, identifying novel treatment regimens for BPD is essential.

Cryptotanshinone (CTS) is a bioactive ingredient extracted from the traditional Chinese medicine *Salvia miltiorrhiza*, which possess the pharmacological activities such as anti-inflammatory (Li et al., 2011; Nagappan et al., 2019), anti-tumour (Dalil et al., 2022), anti-fibrotic (Wang et al., 2022; Wei et al., 2023) and neuroprotective (Mao et al., 2021) effects. Recently, it was reported that CTS showed potent anti-inflammatory properties by modulating macrophage polarization *in vitro* (Ye et al., 2022) and attenuated lipopolysaccharide-induced acute lung injury in mice by inhibiting the TLR4-mediated NF-κB signaling pathway (Tang et al., 2014). In a radiation-induced lung injury model in rats, CTS ameliorated lung injury by regulating TGF-β signaling as well as NOX4 expression (Jiang et al., 2019). However, the effect of CTS in hyperoxia-induced lung injury has not yet been explored.

In this study, we aimed to establish a hyperoxia-induced BPD rat model to study the effects of CTS on lung inflammation and fibrosis. Furthermore, human embryonic lung fibroblast (HFL-1) cells were cultured using post-hyperoxic RAW264.7 cell supernatant to explore the effect of CTS on macrophage expression and fibroblast activation.

## 2 Materials and methods

### 2.1 Chemicals and reagents

CTS (purity ≥ 98%) was obtained from MedChemExpress Co., Ltd., (NJ, United States). Dulbecco’s Modified Eagle’s Medium (DMEM), Ham’s F-12K medium and foetal bovine serum (FBS) were obtained from Gibco (NY, United States). RIPA Lysis Buffer, protease inhibitor, BCA Protein Assay Kit and enhanced chemiluminescence exposure solution were purchased from NCM Biotech (Suzhou, China). TRIzol was obtained from Invitrogen (CA, United States). Antibodies against α-SMA, TGF-β1 and collagen-Ⅰ were obtained from Abcam (Cambridge, MA). GAPDH was obtained from Proteintech (Wuhan, China). Reverse Transcription Kit, Polymerase Chain Reaction Kit and Cell Counting Kit were purchased from Proteinbio (Nanjing, China). TGF-β1, IL-6 and TNF-α ELISA Kit were obtained from Absin (Shanghai, China). Finally, HYP assay kit was purchased from Wuhan Elite Biotechnology Co., Ltd., (Wuhan, China).

### 2.2 Animal model and intervention

Sprague–Dawley (SD) pregnant female rats (SPF grade, 280–300 g) were obtained from Spelford Biotechnology Co., Ltd., (Beijing, Shanghai) and housed in an SPF-grade environment. Each pregnant SD rat was housed individually and underwent delivery spontaneously on the 21st day of pregnancy. The SD rats used in this experiment were all male neonates within 24 h of birth, with a birth weight of 6–8 g. This experiment was approved by the ethics committee of the Affiliated Huaian No. 1 People’s Hospital of Nanjing Medical University (approval number: DW-P-2023-001-10).

**FIGURE 1:**
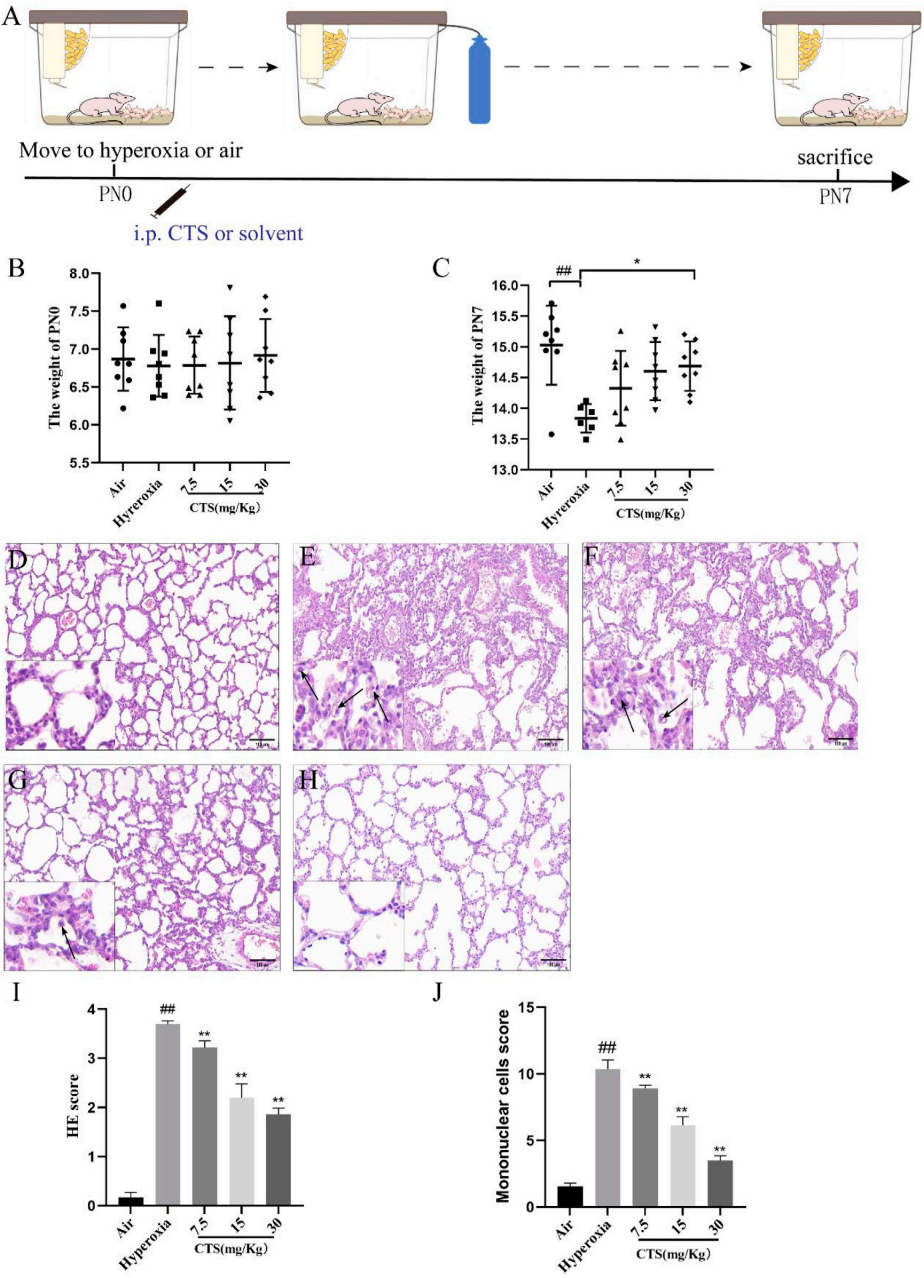
Cryptotanshinone (CTS) protected against hyperoxia-induced lung injury. **(A)** Experimental procedure of BPD model. Weight of the rats at birth **(B)** and postnatal 7 days **(C)**. Haematoxylin and eosin staining of the lung tissue in the **(D)** Air; **(E)** Hyperoxia; **(F)** CTS 7.5 mg/kg; **(G)** CTS 15 mg/kg; and **(H)** CTS 30 mg/kg groups (200×mag, scale bar: 100 μm, black arrows point to mononuclear cells). **(I)** Alveolar injury score, *n* = 6. **(J)** Mononuclear cells score, *n* = 6. The data are presented as the mean ± SEM. ^##^ *p* < 0.01 vs. the air group; *p < 0.05, **p < 0.01 vs. the Hyperoxia group.

A total of 40 newborn SD male rats were assigned into five groups at random (eight rats in each group), namely, the air group, hyperoxia group and three treatment groups with different concentrations of CTS (7.5 mg/kg, 15 mg/kg, and 30 mg/kg) dissolved in a solvent (5% DMSO + 10% Tween-80 + 40% PEG300 + 45% saline), which were administered intraperitoneally. As shown in Figure 1A, the hyperoxia and intervention groups were placed in a hyperoxia chamber from the day of birth, continuously inhaling 95% O_2_ for 7 days. The oxygen concentration analyzer continuously monitored the oxygen concentration in the chamber and soda lime was used to adsorb the exhaled CO_2_ of the rats. Additionally, the air group mice were placed in a normoxic environment (21% O_2_) for 7 days, and other experimental conditions and control factors did not differ from those of the hyperoxia group. During the period from the day of birth to the last day of modelling, CTS was administered daily between 9:00 a.m. and 10:00 a.m. to the treatment groups while the air and hyperoxia groups were injected with equal amounts of solvent. Notably, during the modeling period, clean bedding, supplemental water and food were provided daily, and lactating rats were exchanged between hyperoxic and normoxic environments to prevent hyperoxia toxicity. The rats were euthanised and the lungs were collected for further study at the end of the 7 days. During the experiment, any manipulation that might cause discomfort to the rats was performed with caution. We reduced the level of stimulation and shortened the duration of the experiment to minimize their discomfort, while they were appropriately calmed to reduce fear and anxiety in the rats.

### 2.3 Histopathological evaluation

The left upper lung lobe was fixed in 4% paraformaldehyde overnight, and then the lung tissue was embedded in a wax block and cut into 4 µm thick serial sections. Following this, haematoxylin and eosin (HE) staining and Masson staining were performed at room temperature to determine the extent of alveolar inflammation and fibrosis. Six fields of view were randomly selected for each stained section for observation under a light microscope (magnification, ×40). Ye et al. (2022) assessed the degree of inflammatory damage in the lung using a score based on four parameters: the degree of interstitial oedema, inflammatory cell infiltration, alveolar septal thickness and alveolar congestion level. The score was graded as follows: Level 0 was normal; Level 1 was 25%; Level 2 was 25%–50%; Level 3 was 50%–75%; and Level 4 was ≥75%. Based on previous reports (Xu et al., 2019; Pang et al., 2021), for fibrosis scoring, the degree of fibrosis was graded into three categories: grade 0: no substantial fibrosis throughout the lung; grade 1: mild fibrosis (20%); grade 2: moderate fibrosis (20%–50%); and grade 3: severe fibrosis (>50%). Furthermore, the number of mononuclear cells was also calculated for each group (Ozdemir et al., 2022), and 10 fields of view were randomly selected for counting in each section.

### 2.4 Determination of hydroxyproline (HYP) content

The HYP content in lung tissue was determined using the acid water method according to previous report. A total of 50 mg of lung tissue was first weighed and then added to a glass container after sufficient sizing. Following this, 0.5 ml of 6 mol/L hydrochloric acid solution was added to the glass container and the sample was hydrolysed at 95°C for 6 h. The pH of the sample hydrolysis solution was then adjusted to 6.5–7.0 with final volumn of 5 ml. The sample was then decolourized and the supernatant was collected for HYP determination. The procedure was carried out in accordance with the instructions of the HYP assay kit and the optical density value at 558 nm was measured using a microplate reader (Bio-Tek, Winooski, United States).

### 2.5 RT-qPCR

TRIzol reagent was used to extract total RNA from rat lung tissues and HFL-1 cells, which was then reverse transcribed to cDNA using a Reverse Transcription Kit. Following the instructions of the Polymerase Chain Reaction Kit, 2×SYBR qPCR Mix (12.5 µl), cDNA (1 µl), pre-primer (10 μmol/L; 1 μl) and post primer (10 μmol/L; 1 µl) were mixed and the total volume was made up to 25 µl with ddH_2_O. Finally, the reaction was performed by LightCycler 480 real-time PCR instrument (Roche, United States). Table 1 provides a list of the primer sequences.

**TABLE 1:** A list of the primer sequences.

Species	Gene	Forward (5′-3′)	Reverse (5′-3′)
Rat	GAPDH	CCG​CAT​CTT​CTT​GTG​CAG​TG	TAC​GGC​CAA​ATC​CGT​TCA​CA
	α-SMA	CAT​CCG​ACC​TTG​CTA​ACG​GA	CCA​CAT​ACA​TGG​CAG​GGA​CA
	IL-6	AGA​GAC​TTC​CAG​CCA​GTT​GC	AGT​CTC​CTC​TCC​GGA​CTT​GT
	TNF-α	AGA​AAC​ACA​CGA​GAC​GCT​GA	CAT​TGG​AAT​CCT​TGC​CGG​TG
Human	GAPDH	GGA​GCG​AGA​TCC​CTC​AAA​AT	GGC​TGT​TGT​CAT​ACT​TCT​CAT​GG
	α-SMA	CTA​TGA​GGG​CTA​TGC​CTT​GCC	GCT​CAG​CAG​TAG​TAA​CGA​AGG​A
	MMP2	GAT​ACC​CCT​TTG​ACG​GTA​AGG​A	CCT​TCT​CCC​AAG​GTC​CAT​AGC
	MMP9	GGG​ACG​CAG​ACA​TCG​TCA​TC	TCG​TCA​TCG​TCG​AAA​TGG​GC

### 2.6 Cell culture and treatment

RAW264.7 cells were purchased from the American Type Culture Collection (Rockville, MD, United States). The cells were cultured in a DMEM containing 10% foetal bovine serum and 1% antibiotics. HFL-1 were obtained from Wuxi Newgain Biotechnology Co., Ltd., (Jiangsu, China) and cultured in Ham’s F-12K medium containing 10% foetal bovine serum and 1% antibiotics. Both cells were placed in a constant temperature incubator with 5% CO_2_ at 37°C.

In brief, RAW264.7 cells were seeded in six-well plates at a density of 1 × 10^6^ cells/ml per well with FBS-free medium for 12 h. The cells were pre-treated with CTS for 30 min and then incubated in a hyperoxic incubator at 95% oxygen concentration for 24 h. Finally, the supernatants of these groups were added to the same corresponding groups of HFL-1 for 24 h after the end of modelling.

### 2.7 Cell counting kit-8 (CCK-8) assay

RAW264.7 and HFL-1 cells were seeded into 96-well plates at 5,000 cells per well and incubated overnight at 37°C. Several concentrations of CTS (0, 2.5, 5, 10, and 20 µM) were applied to the cells and incubated for 24 h. The CCK-8 solution was then added in 10 µl to each well, and incubation was carried out for an additional 2 h. Using a microplate reader, the absorbance values of each well were calculated at 450 nm. Each procedure was repeated 3 times.

### 2.8 Enzyme-linked immunosorbent assay (ELISA) detection of cell supernatant

The cell supernatants were further centrifuged and the levels of TGF-β1, IL-6 and TNF-α in the supernatants were measured using ELISA according to the manufacturer’s instructions.

### 2.9 Western blot analysis

Total protein was extracted from HFL-1 cells and rat lung tissue and then quantified, followed by separation in SDS-PAGE and transfer to PVDF membranes (Meck Millipore, Burlington, United States). After 1 h of blocking with 5% milk at room temperature, PVDF membranes were incubated with primary antibodies at 4°C. The following antibodies were used: TGF-β1 (1:1000, Abcam, ab215715), α-SMA (1:2000, Abcam, ab67817), collagen-Ⅰ (1:1000, Abcam, ab260043), and GAPDH (1:3000, Proteintech, 6004-1-ig). The membranes were then incubated with horseradish peroxidase-tagged secondary antibodies at room temperature for 1 h. Finally, protein bands were visualised using an enhanced chemiluminescence exposure solution and quantified using ImageJ analysis software. Three independent samples in each group were tested.

### 2.10 Statistical methods

All data were analysed using the statistical software Graphpad Prism 9.3 (San Diego, CA, United States). The data are expressed as mean ± SEM. One-way analysis of variance was used to compare the data between groups, and Tukey’s *post hoc* test was used to compare the differences between components. *p* < 0.05 or <0.01 denoted a statistically significant difference.

## 3 Results

### 3.1 CTS reduced hyperoxia-induced lung inflammation in SD neonatal rats

As shown in Figures 1B, C, there was no significant difference in the body weight of each group at birth, and the body weight of the hyperoxia group grew slowly and significantly lower than that of the air group after 7 days, and unfortunately 2 rats in the hyperoxia group died during the modeling period. After the CTS intervention, the rats were not only more energetic, but also the body weight of the CTS 30 mg/kg group was significantly higher than that of the hyperoxia group.

HE staining revealed normal alveolar structure, with a clear alveolar wall structure and no obvious inflammatory cell infiltration, in the air group, whereas the lung tissue of SD neonatal rats exposed to hyperoxia showed significant pathological changes, including perivascular oedema and disturbed alveolar structure with a large number of inflammatory cells (shown in Figures 1D, E). However, the degree of alveolar structural damage was reversed after CTS treatment compared to the hyperoxia group, and mononuclear cells were significantly reduced in the intervention group (shown in Figures 1F–H). Alveolar inflammation revealed that the degree of inflammation was more severe in the hyperoxia group compared to the treatment and control groups (shown in Figure 1I). In addition, mononuclear cells were identified via HE staining, and a larger number of mononuclear cells were observed in the hyperoxia group compared with the air group, with an increase in the mean mononuclear cells count score and a significant decrease in the number of mononuclear cells in each group after the intervention (shown in Figure 1J).

### 3.2 CTS attenuated pulmonary fibrosis levels in SD neonatal rats

**FIGURE 2:**
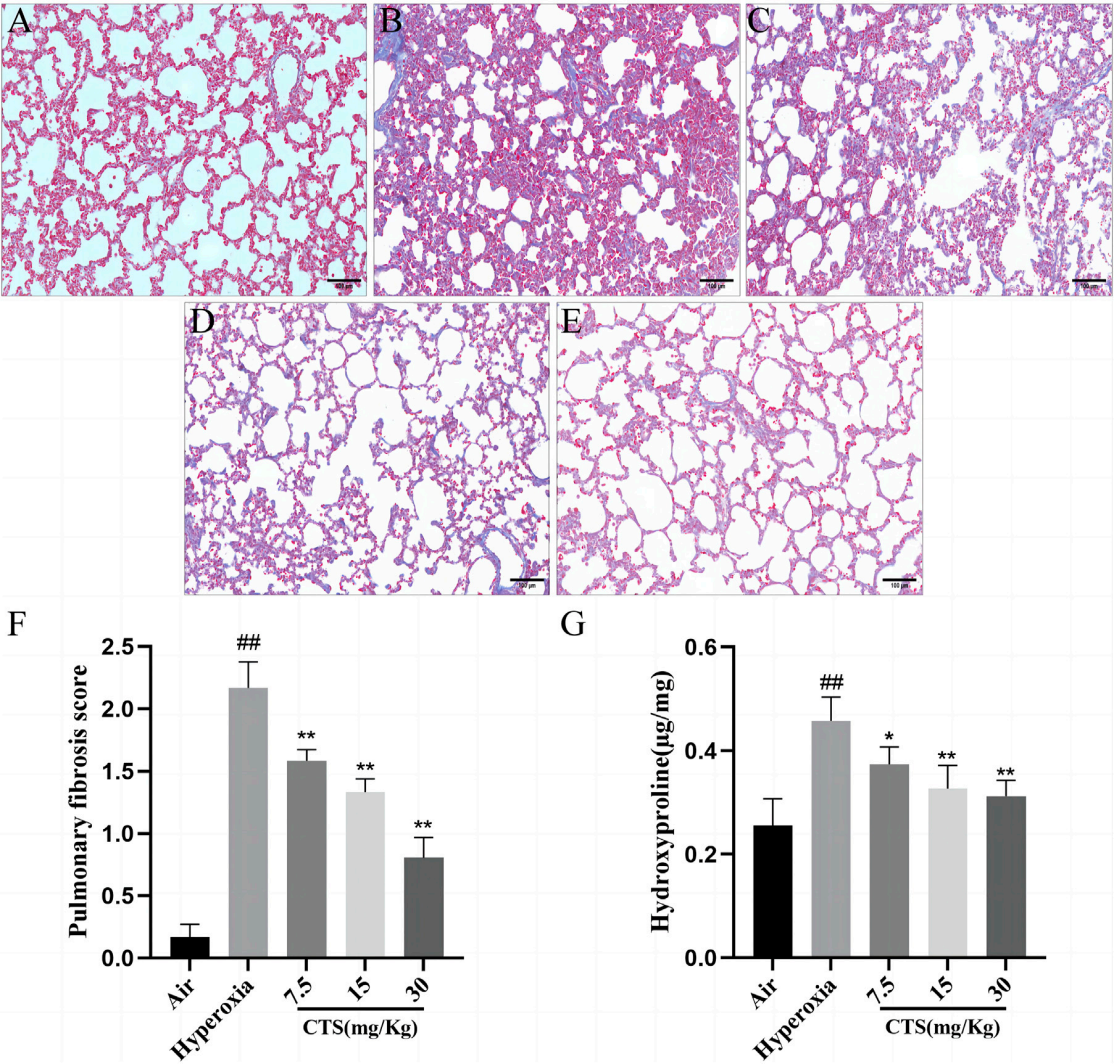
Cryptotanshinone (CTS) reduced hyperoxia-induced collagen accumulation in Sprague–Dawley neonatal rats. Masson staining of the lung tissue in the **(A)** Air; **(B)** Hyperoxia; **(C)** CTS 7.5 mg/kg; **(D)** CTS 15 mg/kg; and **(E)** CTS 30 mg/kg groups (200×mag, scale bar: 100 µm). **(F)** Lung tissue fibrosis score, *n* = 6. **(G)** Lung tissue hydroxyproline content, *n* = 6. The data are presented as the mean ± SEM. ^##^ *p* < 0.01 vs. the air group; *p < 0.05, **p < 0.01 vs. the Hyperoxia group.

As shown in Figures 2A–E, Masson trichrome staining revealed that the lung tissue junction structure of the air group was intact, with the presence of small amounts of blue collagen fibres around the bronchial and alveolar septum regions. Compared with the air group, the hyperoxia group had significantly increased collagen fibre content, irregular arrangement, thickened alveolar septa and increased lung fibrosis with further deposition of collagen fibres. Nonetheless, CTS significantly reduced lung injury and fibrosis, with high doses of CTS exerting the best effect.

Compared with the hyperoxia group, fibrosis was significantly reduced in each intervention group, with the highest reduction observed in the high-dose group (shown in Figure 2F). Notably, HYP is the main component of collagen. Hyperoxia induced a significant increase in HYP content, which gradually decreased after CTS treatment at doses of 7.5–30 mg/kg (shown in Figure 2G).

### 3.3 CTS downregulated TGF-β1 and α-SMA expression in SD rat lungs

**FIGURE 3:**
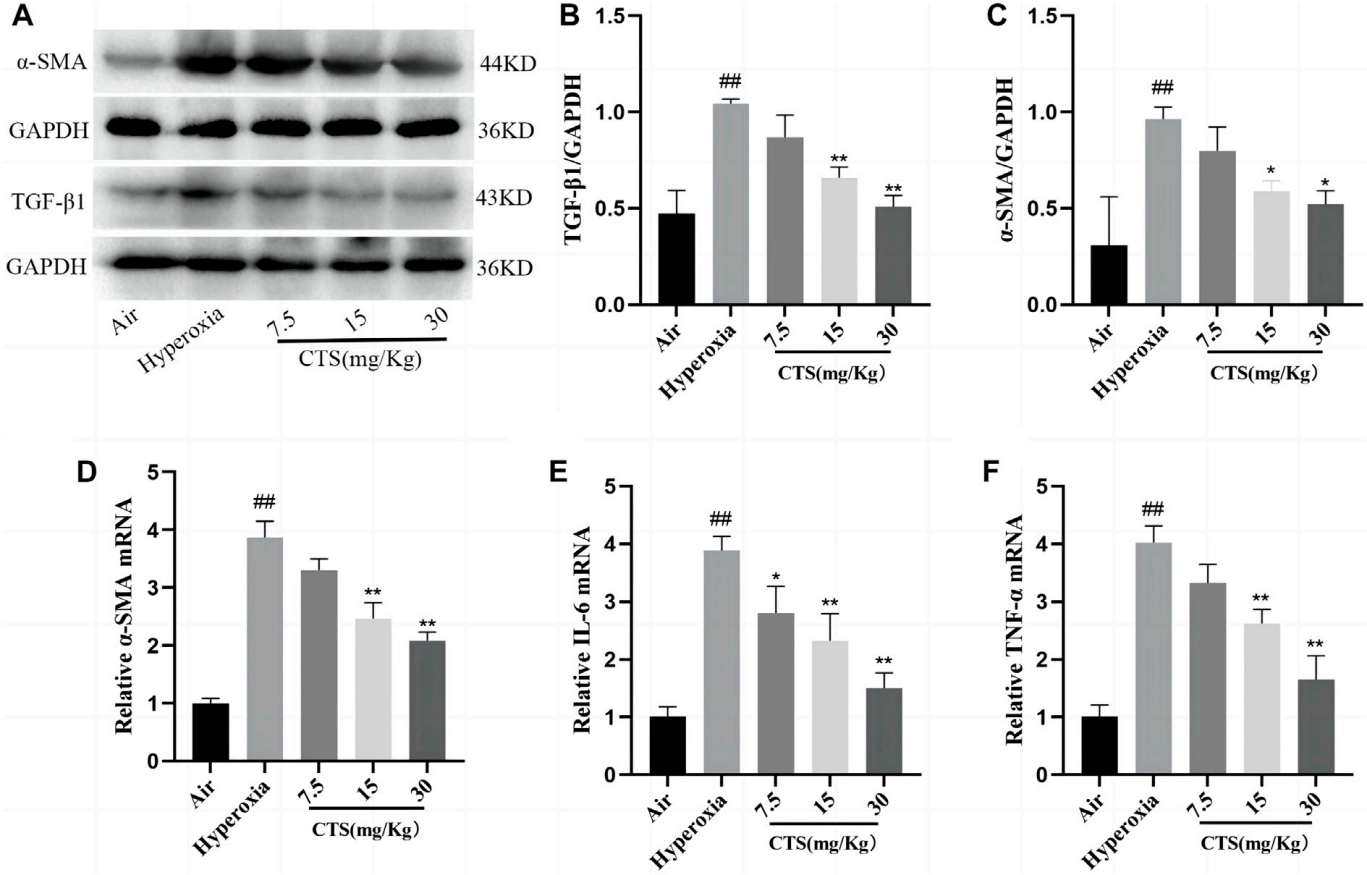
Cryptotanshinone (CTS) reduced the levels of inflammatory and fibrotic factors in the lung tissue of Sprague–Dawley rats. **(A)** Western blot detection of TGF-β1 and α-SMA protein expression levels in mouse lung tissues. **(B)** Relative expression of TGF-β1 protein levels, *n* = 3. **(C)** Relative expression of α-SMA protein levels, *n* = 3. **(D–F)** Relative expression of α-SMA, IL-6 and TNF-α mRNA, *n* = 3. The data are presented as the mean ± SEM. ^##^ *p* < 0.01 vs. air group; *p < 0.05, **p < 0.01 vs. the Hyperoxia group.

Myofibroblasts are characterised by the accumulation of the ECM and high expression of α-SMA, which eventually leads to structural damage and lung tissue dysfunction (Wipff et al., 2007). TGF-β1 is widely used to induce the conversion of fibroblasts to myofibroblasts (Ask et al., 2008). Therefore, we further examined the expression of α-SMA and TGF-β1 in the lung tissue using Western blot. Western blot revealed that after 7 days of exposure to hyperoxia, the expression of α-SMA and TGF-β1 significantly increased in the hyperoxia group and decreased in the different CTS treatment groups (shown in Figures 3A–C). Similarly, α-SMA mRNA expression was significantly increased in the hyperoxia group compared with the air group, but decreased in a dose-dependent manner after the CTS intervention (shown in Figure 3D).

### 3.4 CTS can inhibit the inflammatory cytokines in the lung

After 7 days of hyperoxia exposure, the mRNA expression levels of pro-inflammatory factors IL-6 and TNF-α in the lungs of rats in the model group were significantly increased, and IL-6 and TNF-α in the lungs gradually decreased after CTS intervention, with the most obvious decrease at a dose of 30 mg/kg of CTS (shown in Figures 3E, F).

### 3.5 CTS effectively inhibited the levels of IL-6, TNF-α and TGF-β1 in RAW264.7 cells

**FIGURE 4:**
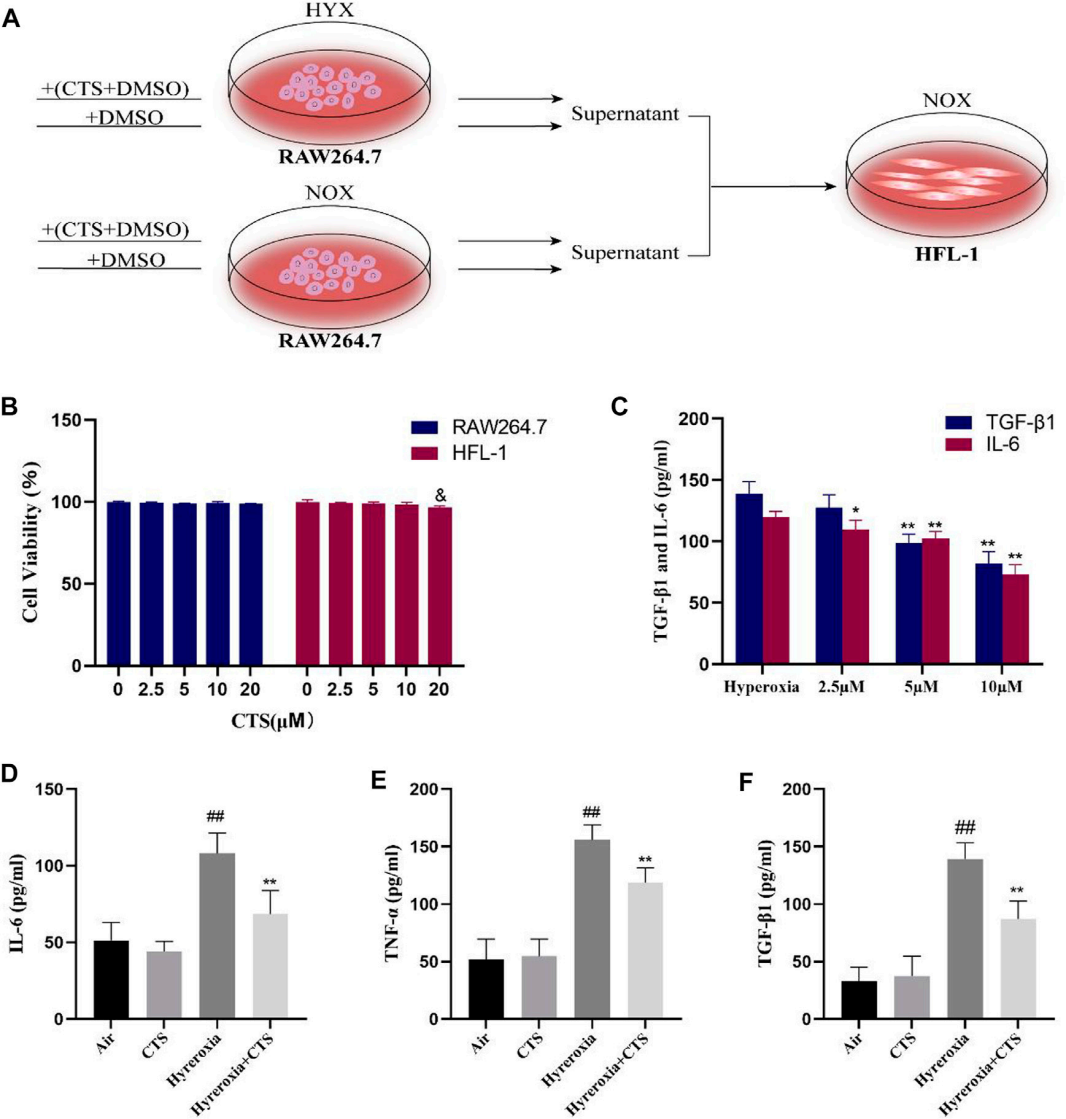
Cryptotanshinone (CTS) inhibited the RAW264.7 cells expression of IL-6, TNF-α and TGF-β levels. **(A)** Model Diagram of HFL-1 co-cultured with the cell suspensions from Raw264.7 exposured to normoxia (NOX, 21% O_2_) or hyperoxia (HYX, 95% O_2_). **(B)** Cell viability was measured using Cell Counting Kit-8 after 24 h of incubation of RAW264.7 and HFL-1 with CTS (0, 2.5, 5, 10, and 20 µM), *n* = 3. **(C)** Levels of TGF-β1 and IL-6 in the supernatant of RAW264.7 cells treated with different concentrations of CTS, *n* = 8. **(D–F)** IL-6, TNF-α and TGF-β1 levels in the supernatant of RAW264.7 cells, *n* = 8. The data are presented as the mean ± SEM. ^##^ *p* < 0.01 vs. the air group; *p < 0.05, **p < 0.01 vs. the Hyperoxia group. &*p* < 0.05 vs. the blank control group (0 µM).

As shown in Figure 4A, we exposed RAW264.7 cells to either normoxic or hyperoxic environments for 24 h, and then added the supernatant to HFL-1 cell culture medium. *In vitro* experiments, we first investigated the viability of CTS on RAW264.7 cells and HFL-1 cells. CTS did not influence cell proliferation at 10 µM concentration for 24 h treatment (shown in Figure 4B), thus, in cellular experiments, we selected 10 µM of CTS for treatment. ELISA of RAW264.7 cell supernatant showed that, in the absence of hyperoxia treatment, CTS at 10 µM did not reduce the levels of IL-6, TNF-α and TGF-β1 in the supernatant. However, the expression of pro-inflammatory factors IL-6, TNF-α and pro-fibrotic factor TGF-β1 was significantly increased after hyperoxia treatment and decreased after CTS treatment (shown in Figures 4D–F). We also found that TGF-β1 and IL-6 in the supernatant showed a dose-dependent decrease after CTS treatment (shown in Figure 4C).

### 3.6 CTS reduced the levels of α-SMA, collagen-Ⅰ and MMPs in HFL-1 cells after treatment with RAW264.7 cell supernatant

**FIGURE 5:**
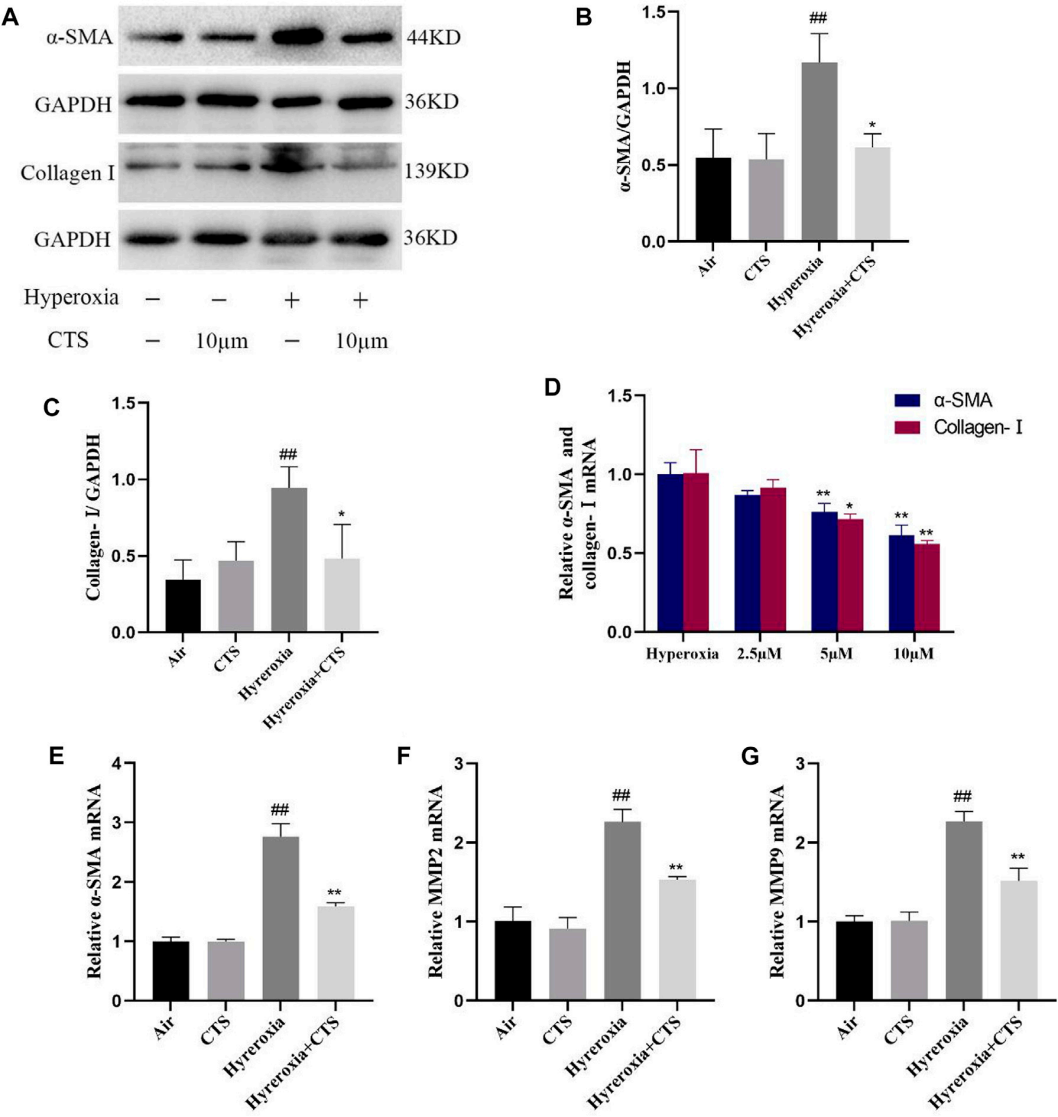
Cryptotanshinone (CTS) further inhibited HFL-1 cells proliferation and differentiation through RAW264.7 cells. **(A)** α-SMA and collagen-Ⅰ protein levels in HFL-1 cells. **(B)** Relative expression of α-SMA protein, *n* = 3. **(C)** Relative expression of the collagen-Ⅰ protein, *n* = 3. **(D)** Relative mRNA expression of α-SMA and collagen-Ⅰ, *n* = 3. **(E–G)** Relative mRNA expression of α-SMA, MMP2 and MMP9, *n* = 3. The data are presented as the mean ± SEM. ^##^ *p* < 0.01 vs. the air group; *p < 0.05, **p < 0.01 vs. the Hyperoxia group.

The immunoblotting results and their grey value analysis demonstrated that there were no significant differences in the myofibroblast biomarkers α-SMA and ECM protein (collagen-Ⅰ) in the blank control and CTS control groups. Moreover, the protein levels of α-SMA and collagen-Ⅰ increased in the hyperoxia group but decreased after CTS intervention (shown in Figure 5A–C). As shown in Figure 5D, the mRNA levels of α-SMA and collagen-Ⅰ were reduced in a dose-dependent manner after CTS treatment, with the most pronounced effect after 10 µM treatment. Similarly, RT-qPCR analysis revealed that the relative mRNA expression of α-SMA was significantly higher in HFL-1 cells after co-culturing with hyperoxic RAW264.7 cell supernatants and lower after CTS intervention (shown in Figure 5E). Matrix metalloproteinases (MMPs) are zinc-dependent endopeptidases that belong to the metzincins protease superfamily and play a key role in the homeostasis of the ECM (Chuliá-Peris et al., 2022). Therefore, we examined the levels of MMP2 and MMP9 and observed an increase in the hyperoxia group and a decrease after CTS intervention (shown in Figures 5F, G). Thus, these findings indicate that CTS can further inhibit HFL-1 proliferation and differentiation by suppressing cytokine expression through RAW264.7 cells.

## 4 Discussion

Various studies have demonstrated that persistent exposure to hyperoxia causes damage to the lungs, leading to BPD (Balasubramaniam et al., 2007). BPD is characterized by inflammation and abnormal lung repair, further leading to fibrosis (Stone et al., 2016; Jia et al., 2021), which can have various adverse effects on the affected children, who are also more prone to respiratory disease in adulthood than others. However, effective therapeutic agents for BPD treatment are scarce (Poets and Lorenz, 2018). Previous studies have shown that CTS plays an important role in lung injury (Jiang et al., 2019; Ye et al., 2022), and the anti-inflammatory and anti-fibrotic effects of CTS are gradually being explored (Li et al., 2021). Accordingly, this study aimed to explore the anti-inflammatory and anti-fibrotic effects of CTS in a BPD model.

In the early stage, inflammatory responses and cytokine dysregulation are the main features of BPD. Previous studies have demonstrated that alveolar macrophages increase significantly after hyperoxia treatment and are involved in the development of abnormal structures in the immature mouse lung (Bhandari et al., 2006; Liao et al., 2015; Kalymbetova et al., 2018), suggesting that macrophages are participated in tissue development, physiological regulation, and immune function *in vivo* (Pollard, 2009; Wynn et al., 2013). Notably, after birth, macrophages generated from the yolk sac can colonize the alveolar cavity and develop further in the distal lung (Guilliams et al., 2013; Jones et al., 2013). Under the pathological conditions of BPD, monocytes from the bone marrow can be recruited into the lungs and differentiate into alveolar macrophages to participate in the inflammatory process (Misharin et al., 2017; Wang and Tsao, 2020; Martin et al., 2021). To investigate the response of CTS to hyperoxia-induced lung inflammation, this study, like other studies using animal hyperoxia models, focused on the pathological assessment of lung inflammation. Exposure to hyperoxia for 7 days resulted in significant alveolar damage and visible mononuclear cells infiltration. Additionally, histological scoring indicated that CTS was effective in reducing alveolar damage scores and decreasing mononuclear cells infiltration.

In the late phase of BPD, macrophages play a pivotal role in the pathogenesis of pulmonary fibrosis as the main effector cells of pulmonary fibrosis (Liu et al., 2016; Zhang et al., 2018; Du et al., 2019). TGF-β1 is both an important pro-inflammatory factor and a critical pro-fibrotic factor in the lung (Keenan et al., 2018). Macrophages can promote fibroblast activation through TGF-β1 alone or synergistically with inflammatory factors to drive fibroblast activation, induce fibroblast proliferation and differentiation and promote collagen synthesis while aggregating multiple inflammatory cells and promoting inflammatory factor release (Lodyga and Hinz, 2020; Su et al., 2020; Bolourani et al., 2021). In a hyperoxia-induced animal model, we examined the levels of these growth factors using Western blot and RT-qPCR techniques. The exposure to hyperoxia significantly increased the levels of the fibrogenic growth factor TGF-β1 and the inflammatory factors IL-6 and TNF-α, which were significantly downregulated after CTS intervention. Fibroblasts also play an pivotal role in the development of BPD, as fibroblasts are speculated to respond to environmental stress through paracrine signals that drive their proliferation, differentiation into myofibroblasts and migration into the alveolar septum and the ECM (Sucre et al., 2016; Gao et al., 2019). These functions consequently exacerbate fibrosis in the lung. Additionally, we examined the expression of the myofibroblast marker α-SMA in the lung tissue, wherein α-SMA protein level was significantly elevated after hyperoxia and reduced after CTS intervention.

In lung tissue, alveolar macrophages are the immune cells that are most prevalent, and activation of the pro-inflammatory phenotype plays a key role in lung injury by releasing pro-inflammatory factors to stimulate cytokine storms (Zhou et al., 2020). *In vivo*, CTS treatment significantly reduced the levels of inflammatory factors and fibrotic factors in hyperoxia-induced SD neonatal rat lungs. Considering that macrophages are the main source of these factors, we examined the levels of cytokines secreted by RAW264.7 cells in a hyperoxia culture *in vitro*, which revealed consistent results with that of the *in vivo* experiments. Moreover, IL-6, TNF-α and TGF-β1 levels were increased under hyperoxic conditions but decreased significantly after CTS intervention. TGF-β1 from macrophages or other cells can serve as an accessory signal for macrophage-fibroblast interactions in fibrosis (Buechler et al., 2021), TGF-β1 can further stimulate fibroblast proliferation and migration and promote the uncontrolled differentiation of fibroblasts into myofibroblasts, leading to excessive ECM deposition, including α-SMA and collagen-Ⅰ deposition (Enzo et al., 2019; Zhang et al., 2019). Therefore, the fibroblasts cultured with the corresponding supernatant of macrophages were used for analysis, wherein the levels of α-SMA, collagen-Ⅰ and MMPs expressed by fibroblasts were further reduced. Thus, these findings indicate that CTS exerts anti-inflammatory and anti-fibrotic effects through macrophages in the BPD model.

TGF-β target genes are increasingly considered as causative factors of BPD (Noe et al., 2019). TGF-β1 produced by macrophages can activate the downstream Smad3 pathway via TGFβ receptor 1 subunit/ALK5, inducing activated fibroblasts to express α-SMA and collagen-Ⅰ (Frangogiannis, 2020). LDL receptor-related protein 1 in macrophages can bind to proinflammatory proteins such as TGF-β2 and mediates their endocytosis, leading to a proinflammatory state (Jaeschke and Hui, 2021). Activated macrophages also produce factors that drive fibroblast proliferation, such as platelet-derived growth factors (PDGFs), amphiregulin (AREG), and IL-6 (Buechler et al., 2021). However, the downstream pathway was not explored further in this paper, which will be the direction of our future research.

In conclusion, this study demonstrated that CTS can significantly reduce the degree of alveolar inflammation and fibrosis induced by hyperoxia in SD rats. Additionally, it can also reduce the expression of pro-inflammatory and pro-fibrotic factors via macrophages and thus improve inflammation and fibrosis. Therefore, CTS has the potential as a natural compound for protection against BPD. However, in-depth studies are needed to verify its clinical application.

## Data Availability

The original contributions presented in the study are included in the article/Supplementary Material, further inquiries can be directed to the corresponding authors.
