# Supplementary figures and images for: Cryptotanshinone affects HFL-1 cells proliferation by inhibiting cytokines secretion in RAW264.7 cells and ameliorates inflammation and fibrosis in newborn rats with hyperoxia induced lung injury
Source: Front Pharmacol. 2023 Jul 25;14:1192370. doi: 10.3389/fphar.2023.1192370 (PMC10407416; doi:10.3389/fphar.2023.1192370)

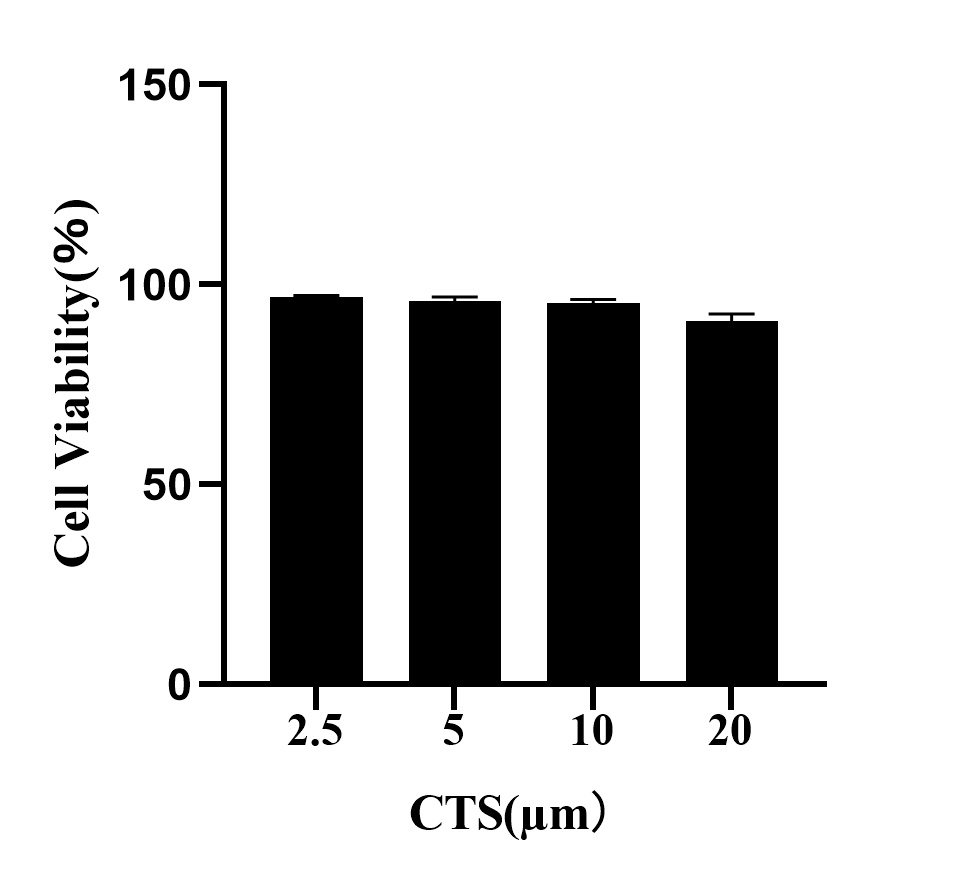

Supplement: Supplementary file 2 [file DataSheet1.ZIP › figures/CCK8/HFL-CCK8.tif]

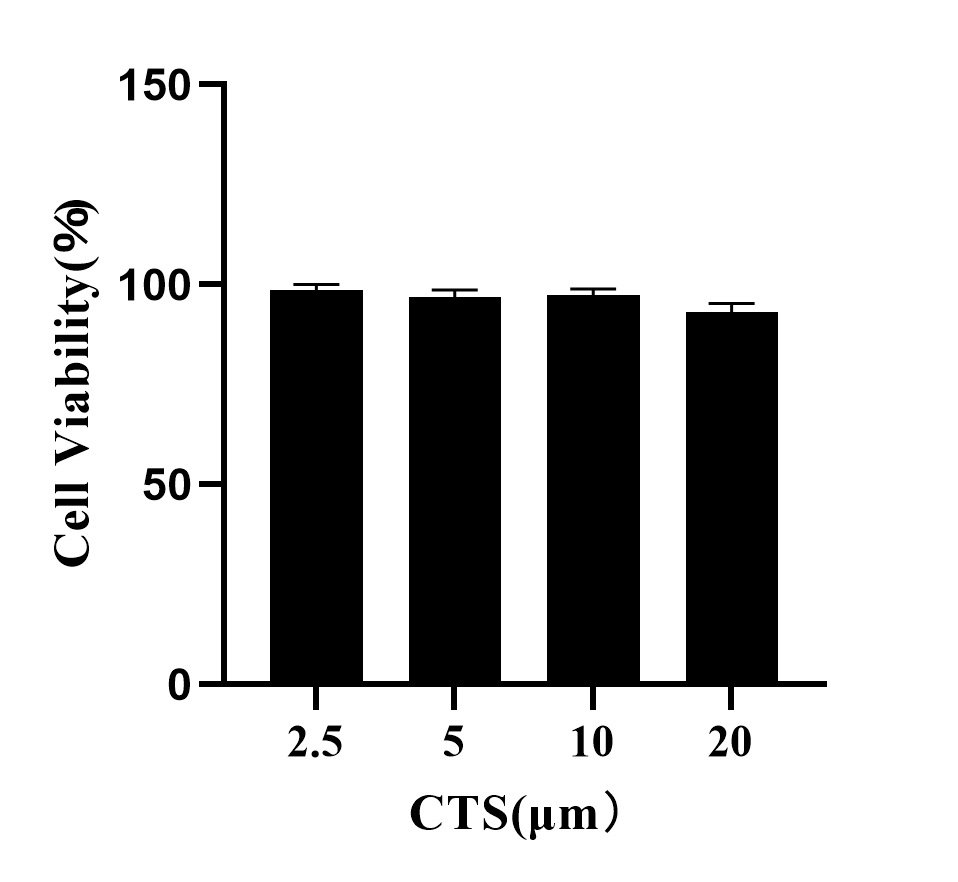

Supplement: Supplementary file 2 [file DataSheet1.ZIP › figures/CCK8/RAW-CCK8.tif]

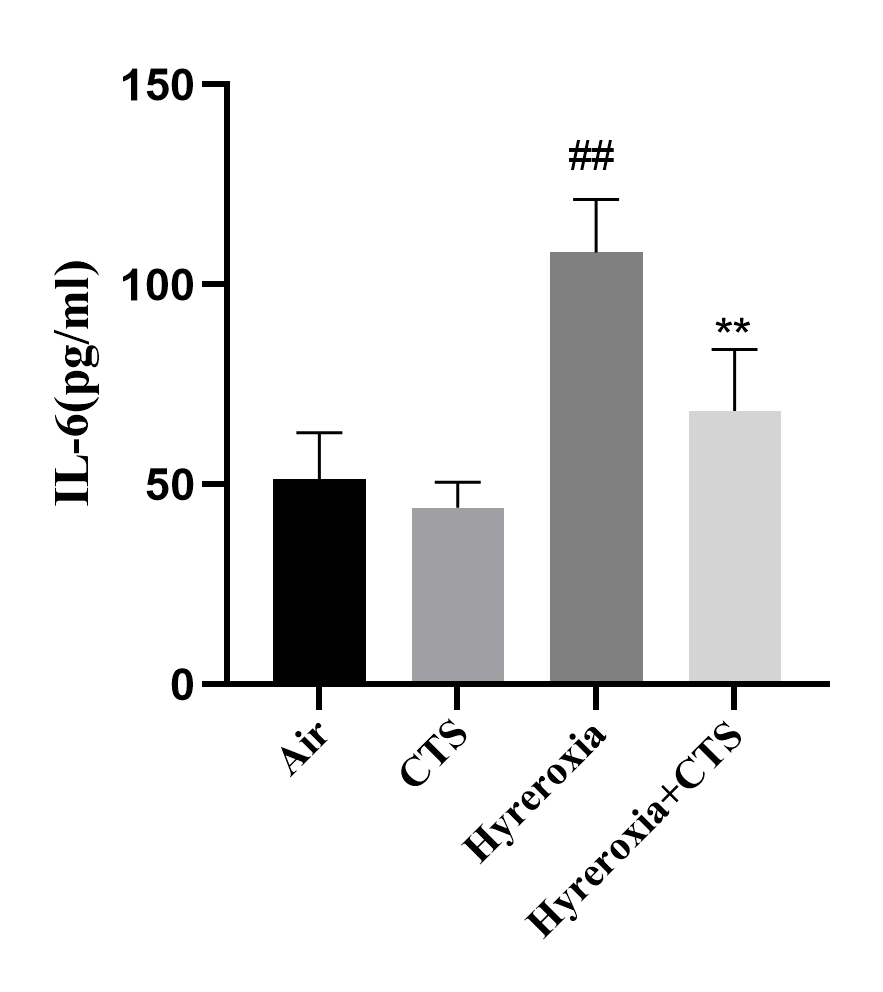

Supplement: Supplementary file 2 [file DataSheet1.ZIP › figures/ELISA/IL6 ELISA.tif]

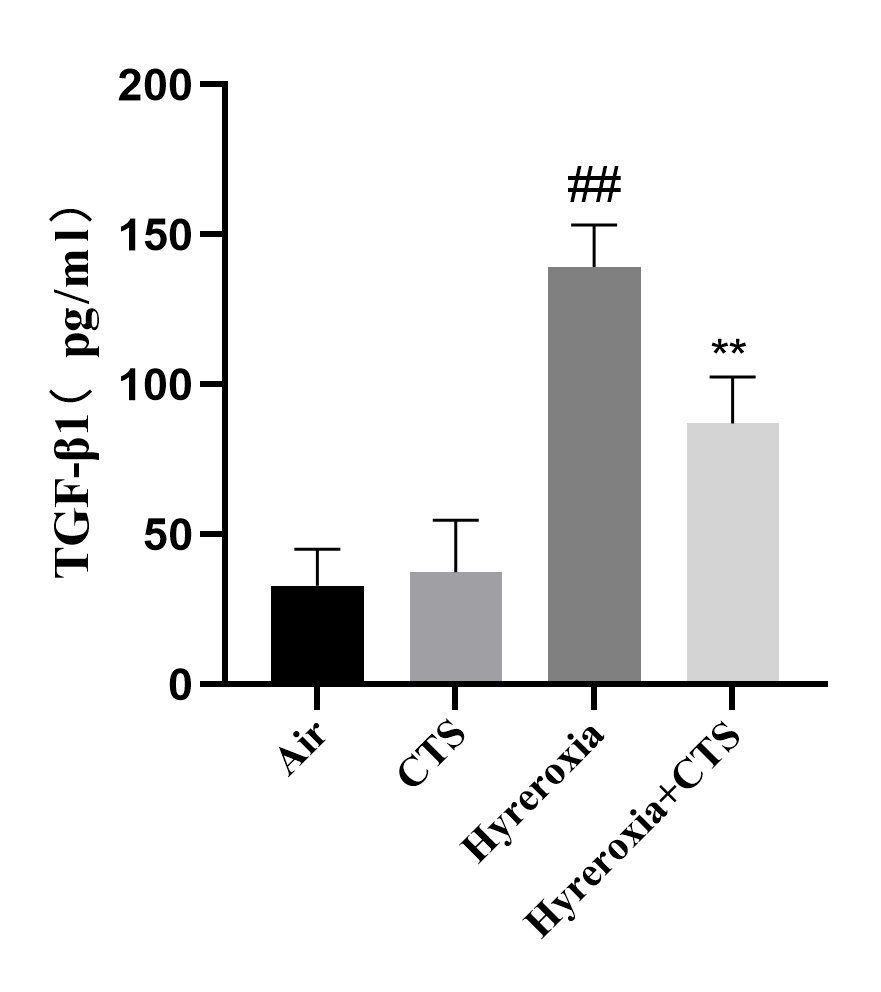

Supplement: Supplementary file 2 [file DataSheet1.ZIP › figures/ELISA/TGF-a┬1.tif]

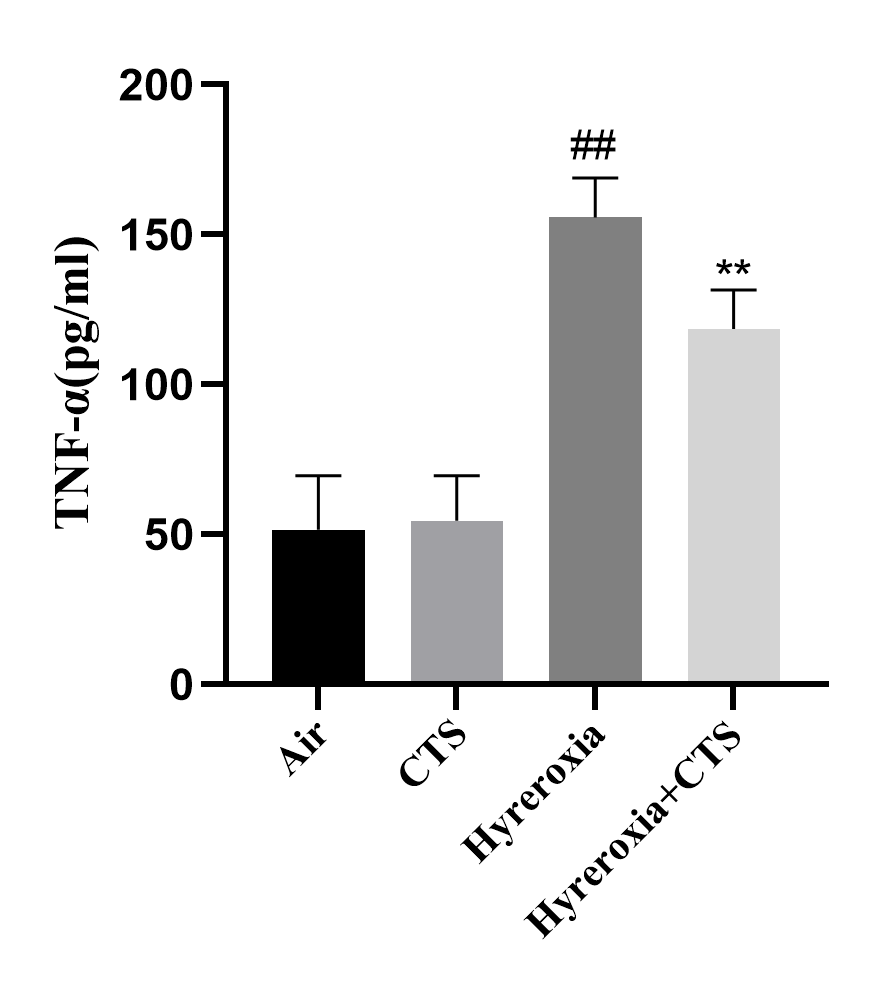

Supplement: Supplementary file 2 [file DataSheet1.ZIP › figures/ELISA/TNF-a┴.tif]

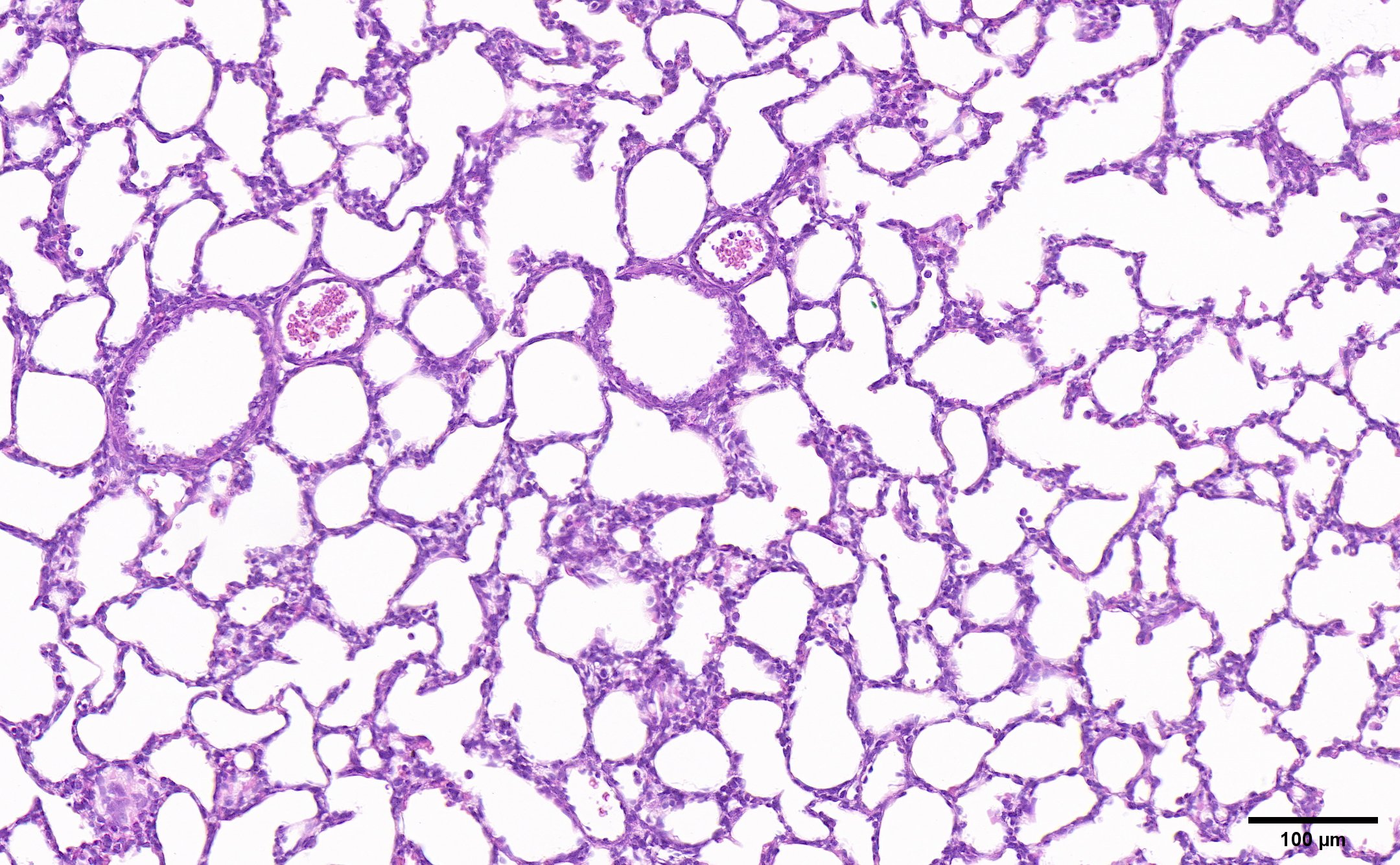

Supplement: Supplementary file 2 [file DataSheet1.ZIP › figures/HE Masson/HE Air.jpg]

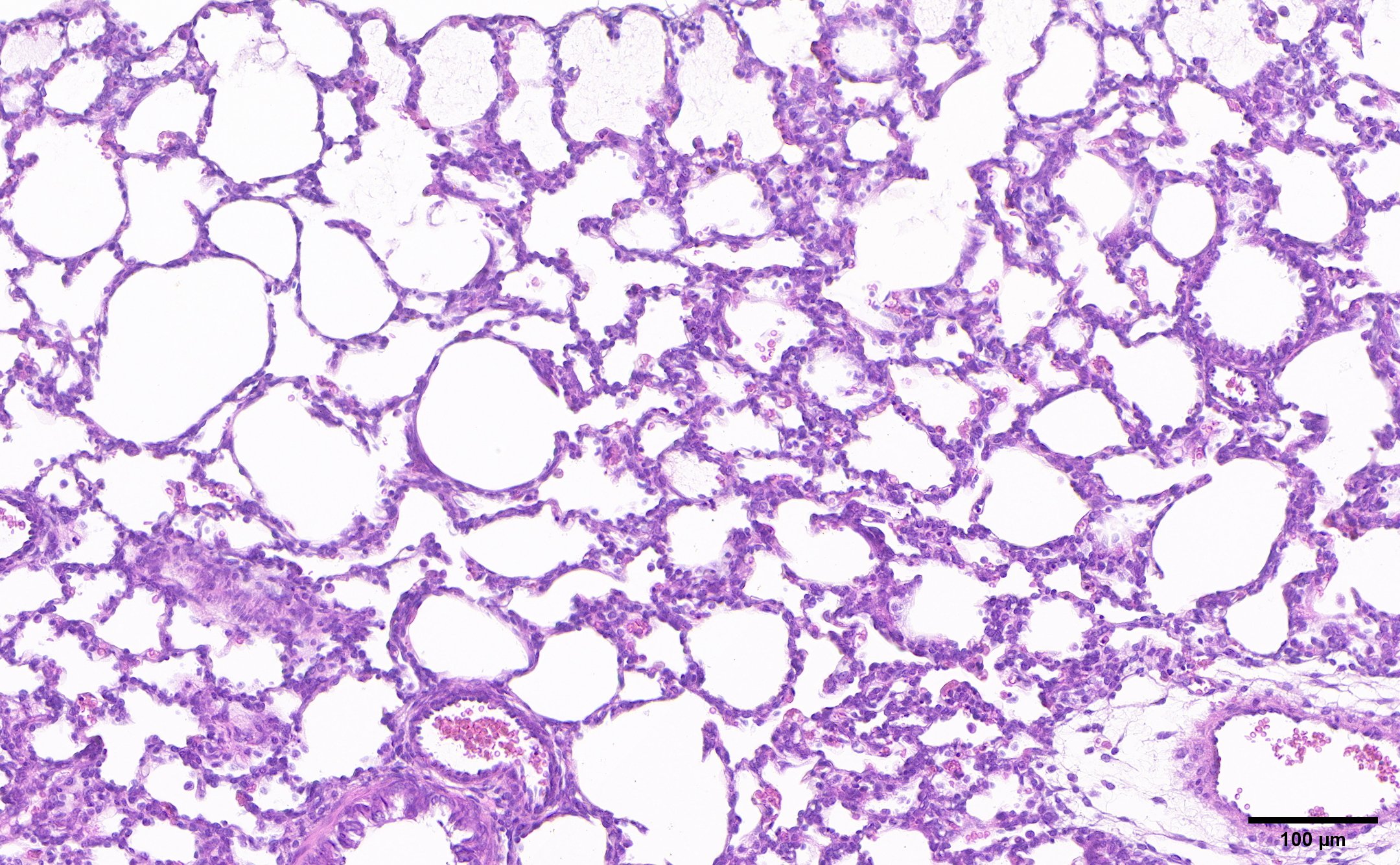

Supplement: Supplementary file 2 [file DataSheet1.ZIP › figures/HE Masson/HE CTS15.jpg]

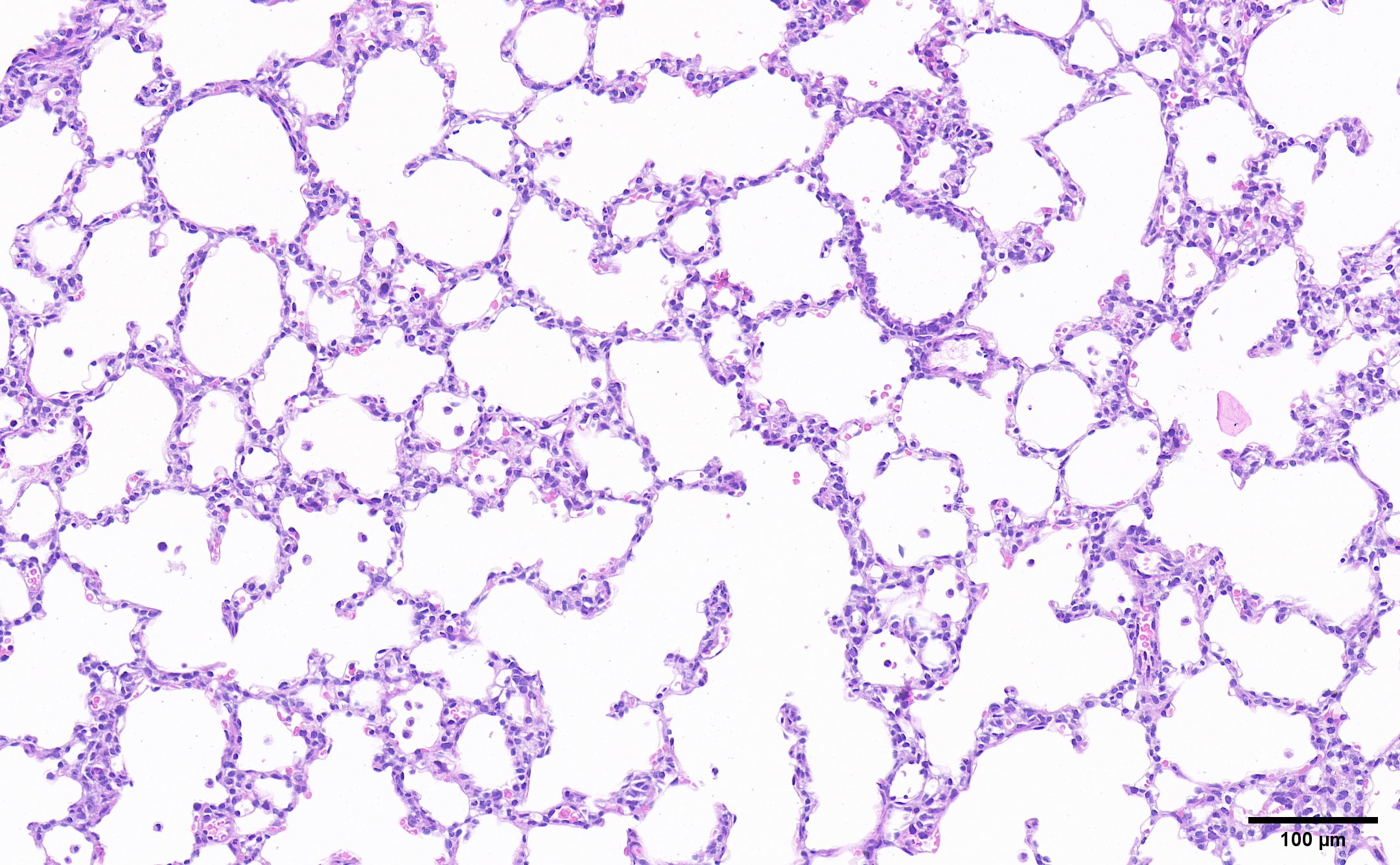

Supplement: Supplementary file 2 [file DataSheet1.ZIP › figures/HE Masson/HE CTS30.jpg]

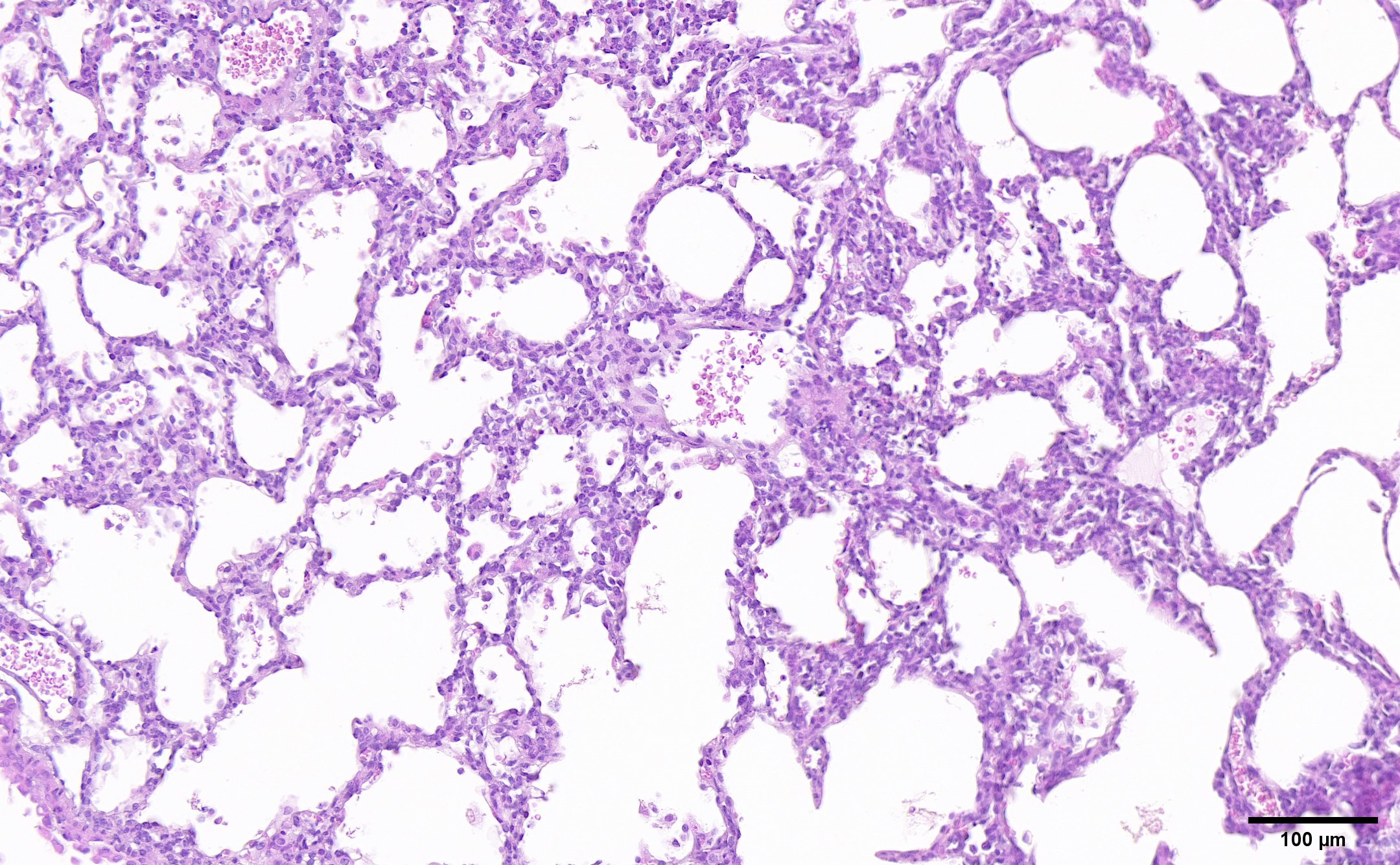

Supplement: Supplementary file 2 [file DataSheet1.ZIP › figures/HE Masson/HE CTS7.5.jpg]

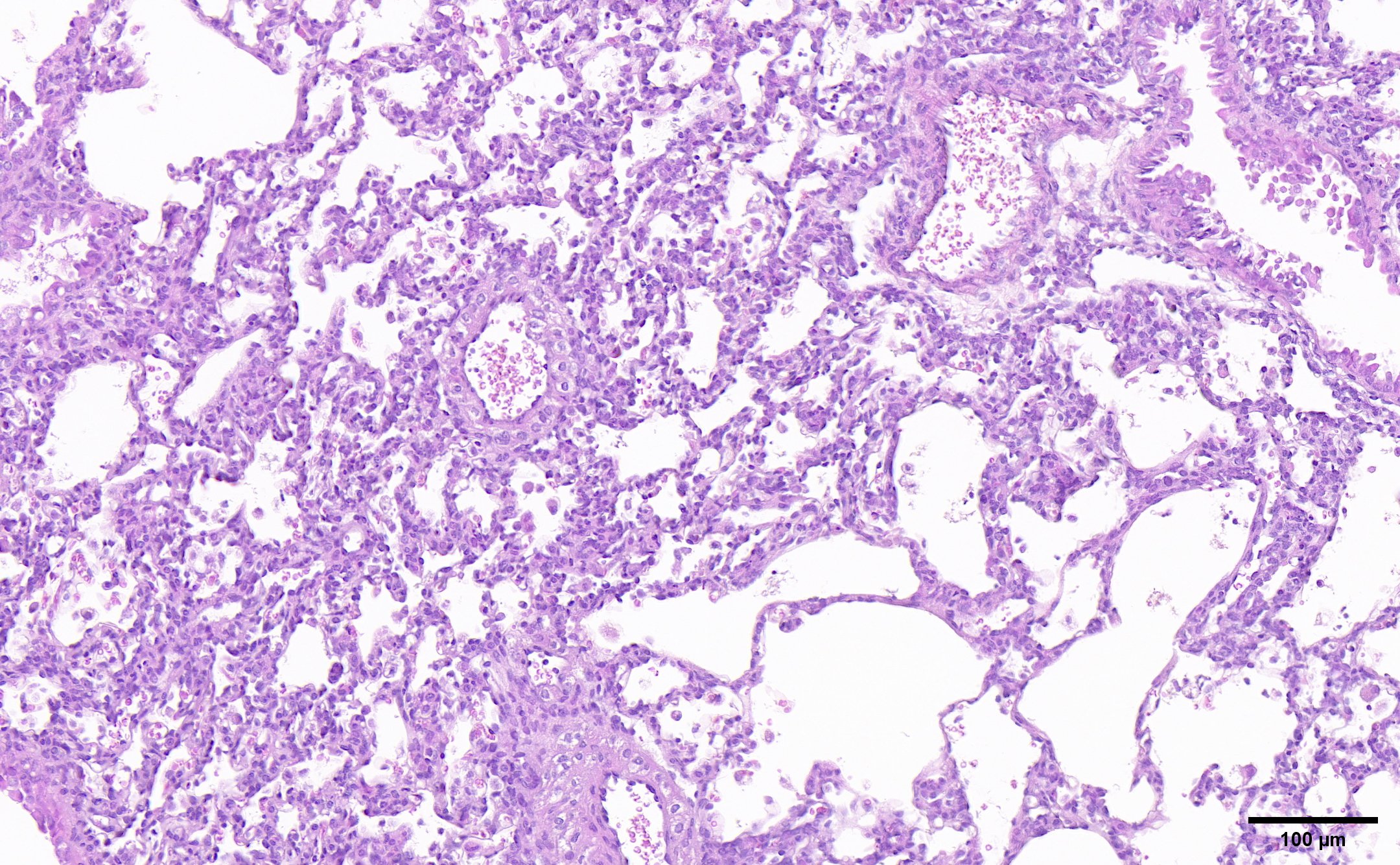

Supplement: Supplementary file 2 [file DataSheet1.ZIP › figures/HE Masson/HE Hyperoxia.jpg]

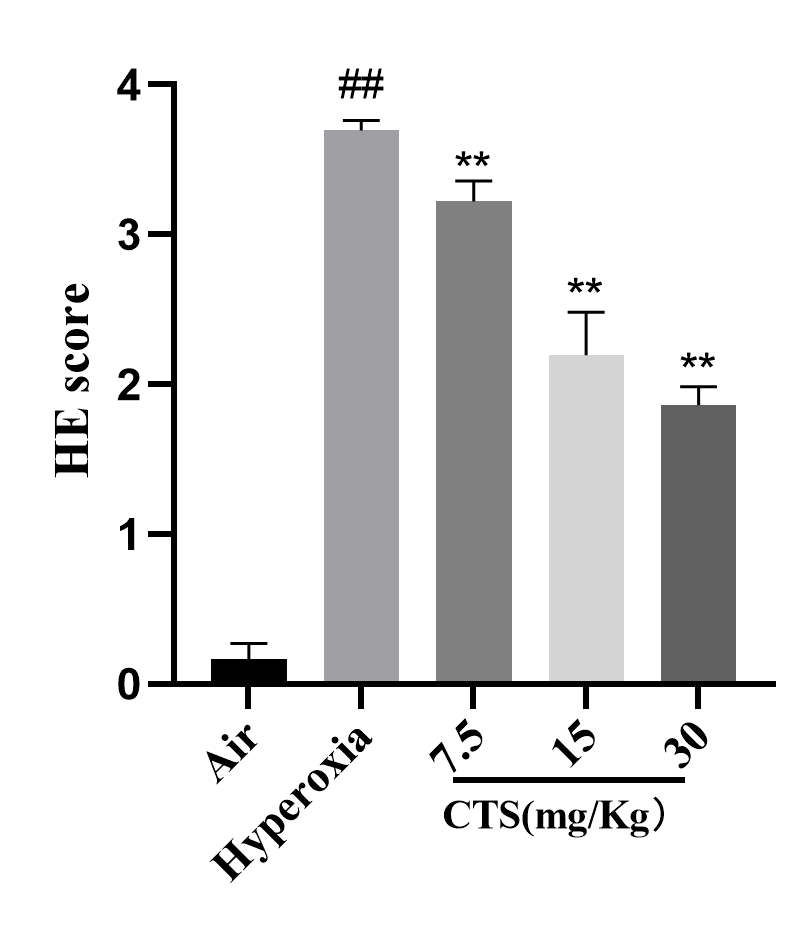

Supplement: Supplementary file 2 [file DataSheet1.ZIP › figures/HE Masson/HE╞└╖╓.tif]

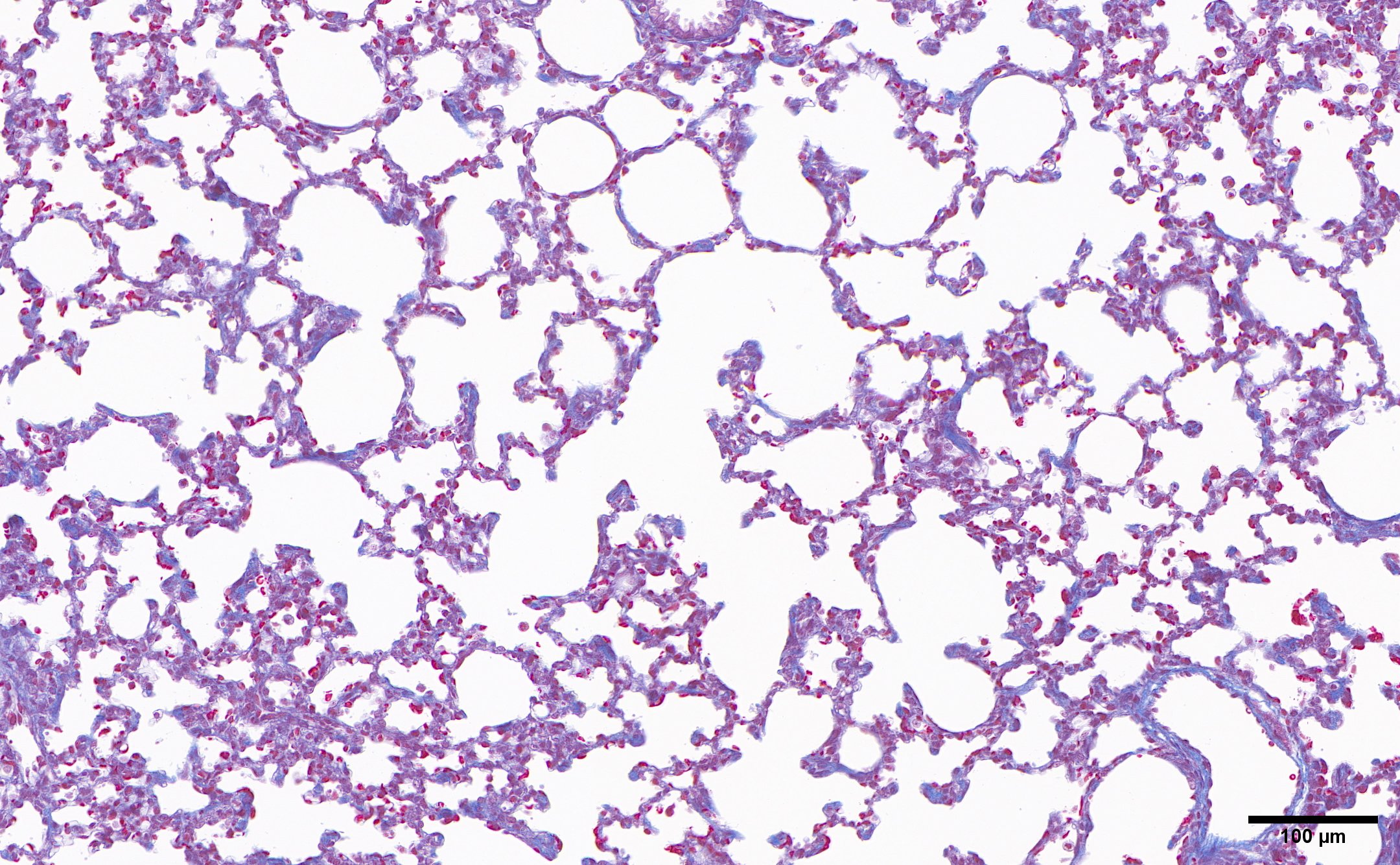

Supplement: Supplementary file 2 [file DataSheet1.ZIP › figures/HE Masson/Masson CTS15.jpg]

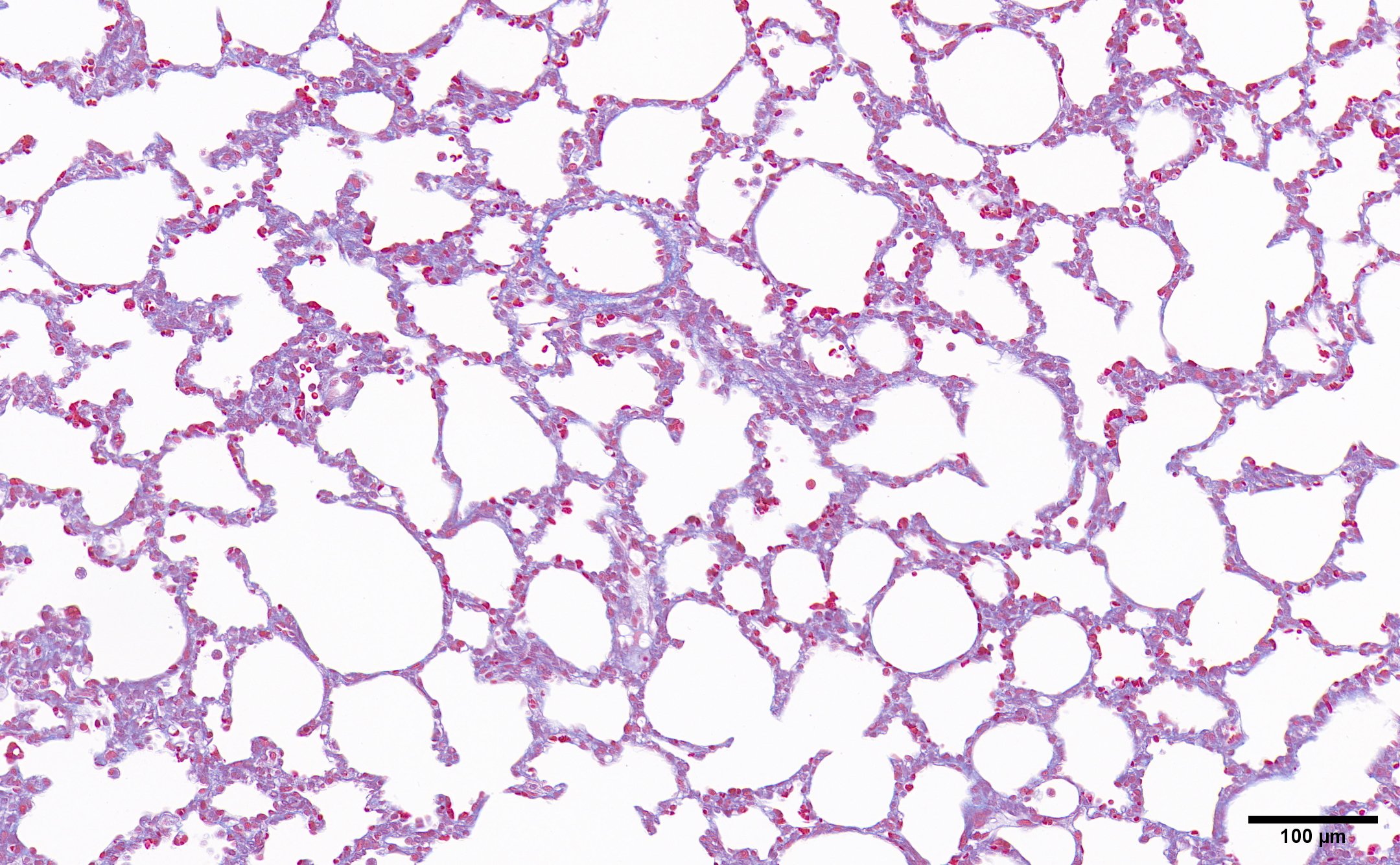

Supplement: Supplementary file 2 [file DataSheet1.ZIP › figures/HE Masson/Masson CTS30.jpg]

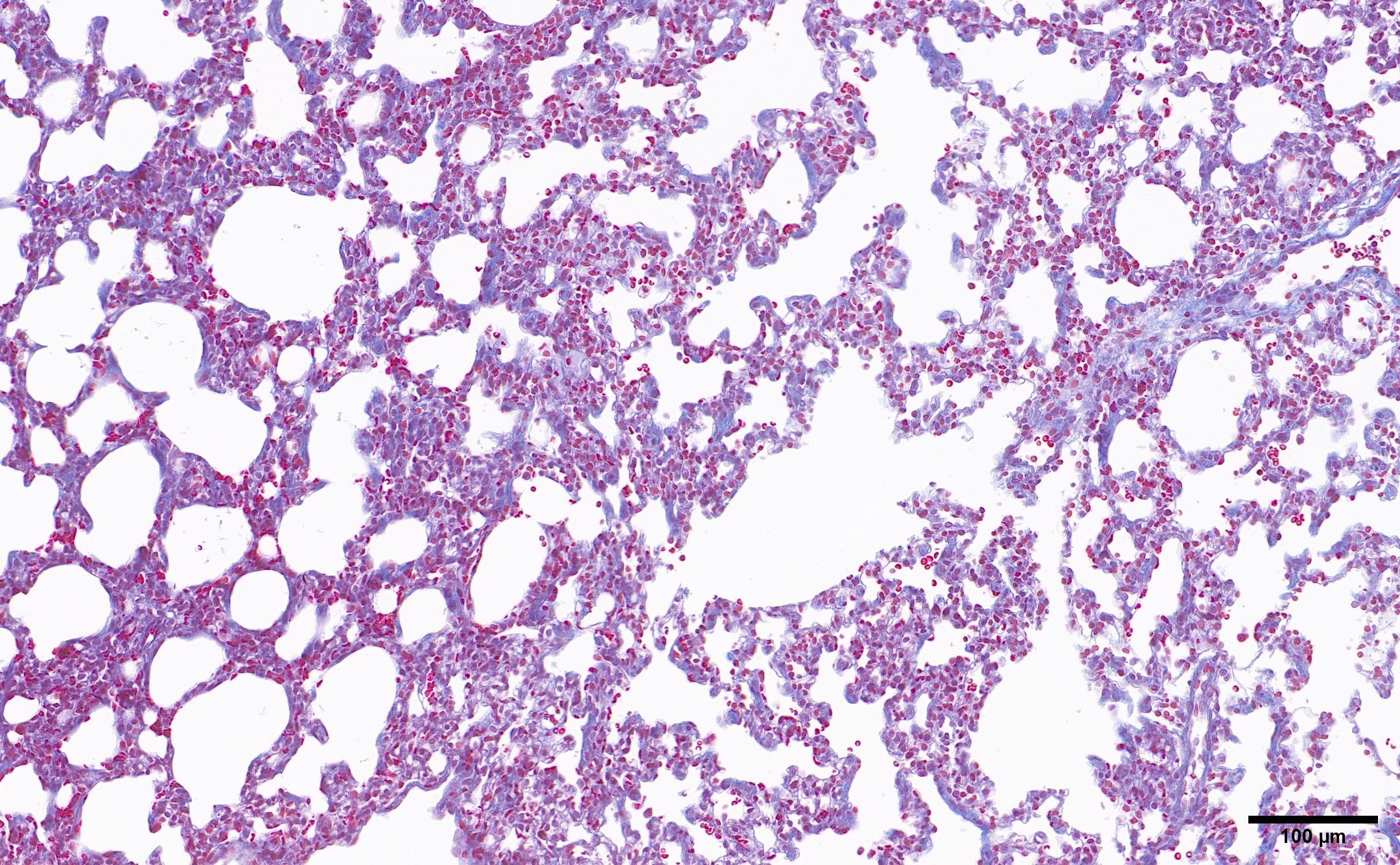

Supplement: Supplementary file 2 [file DataSheet1.ZIP › figures/HE Masson/Masson CTS7.5.jpg]

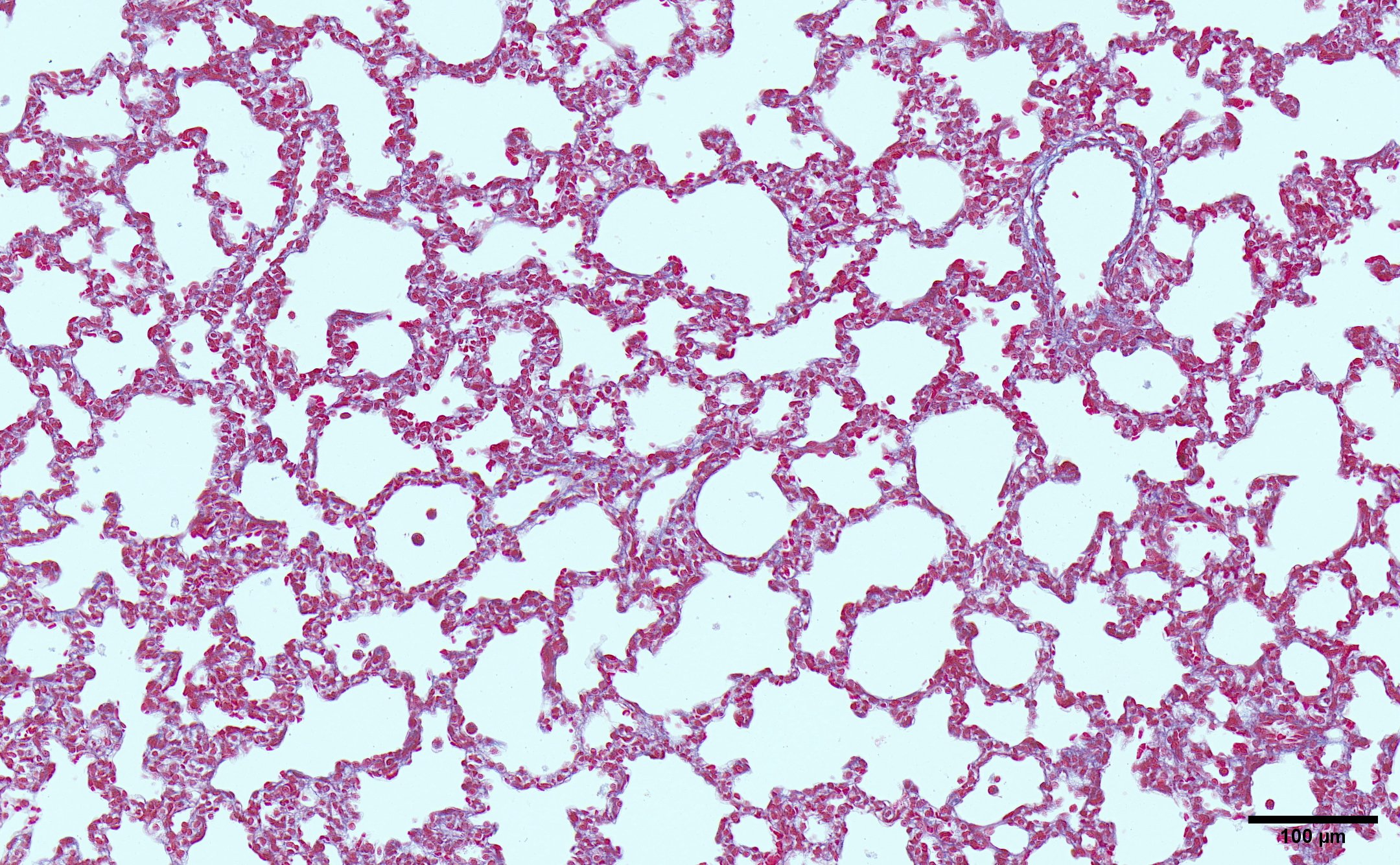

Supplement: Supplementary file 2 [file DataSheet1.ZIP › figures/HE Masson/masson_Air.jpg]

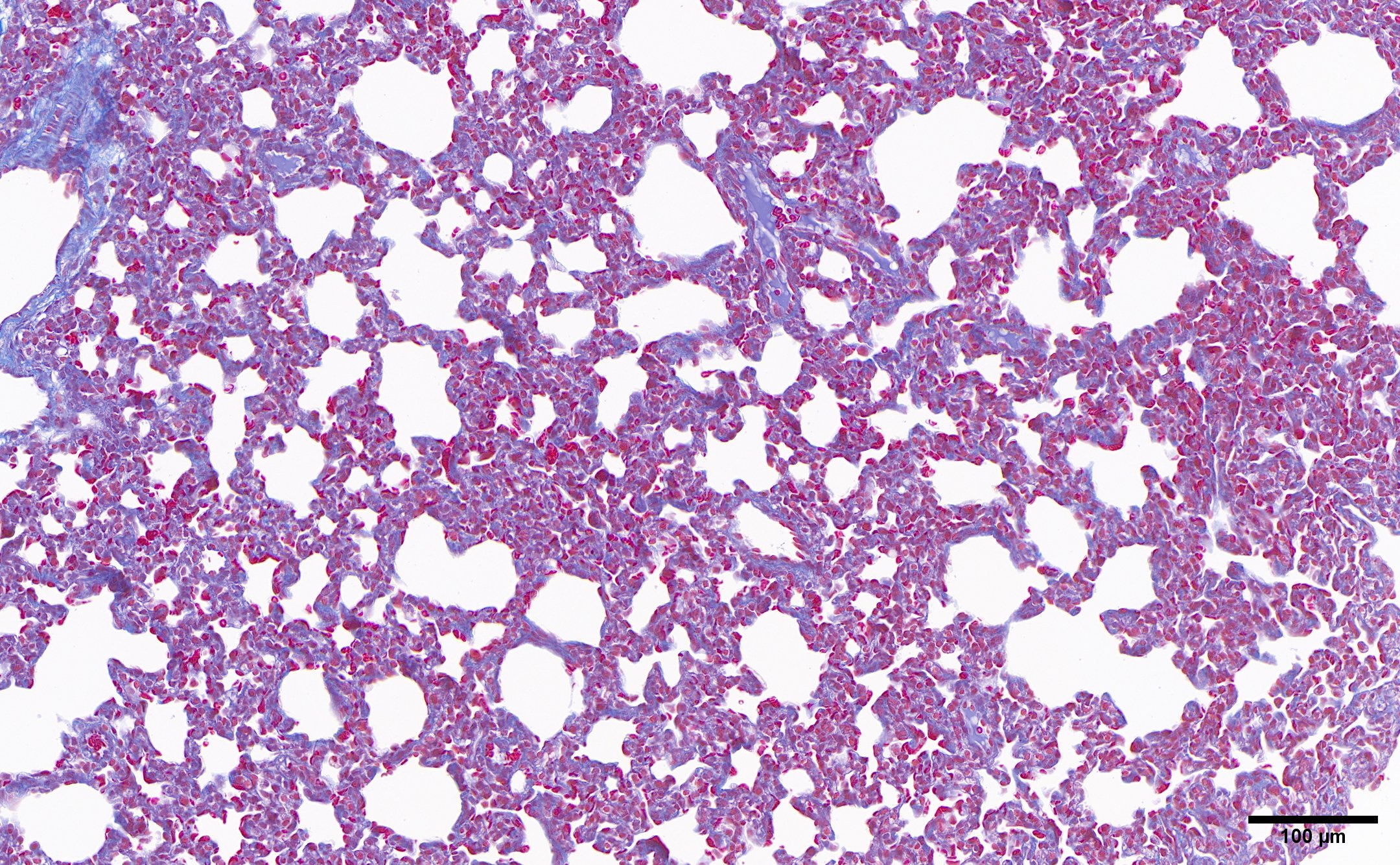

Supplement: Supplementary file 2 [file DataSheet1.ZIP › figures/HE Masson/Masson_Hyperoxia.jpg]

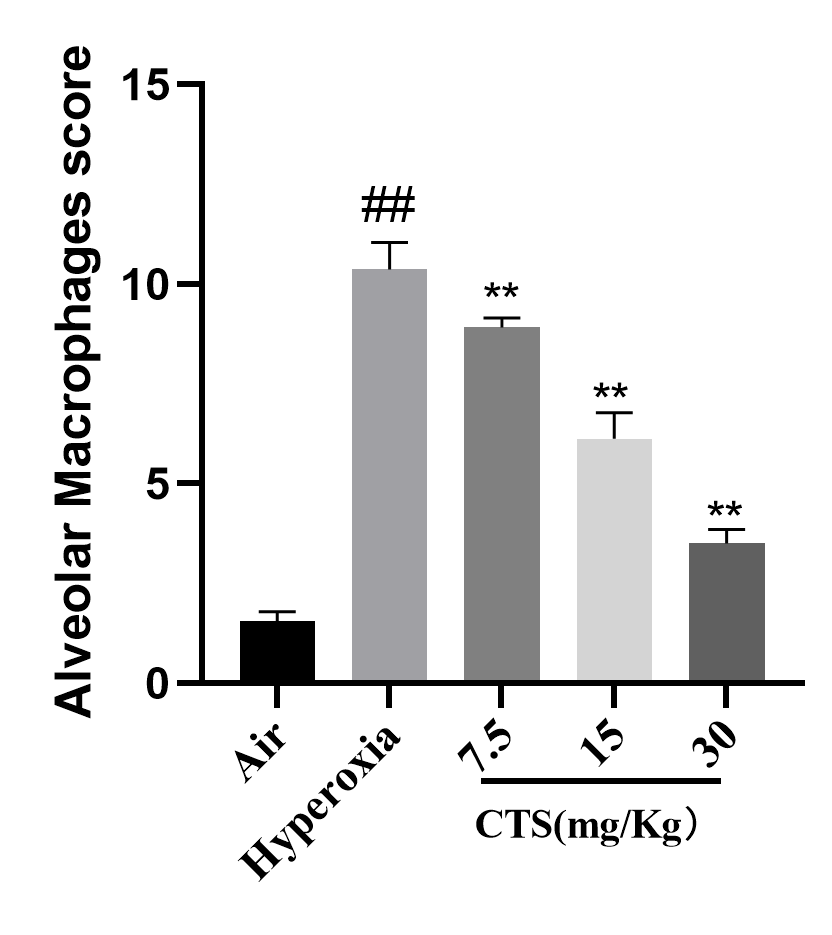

Supplement: Supplementary file 2 [file DataSheet1.ZIP › figures/HE Masson/╛▐╩╔╧╕░√╩2─┐.tif]

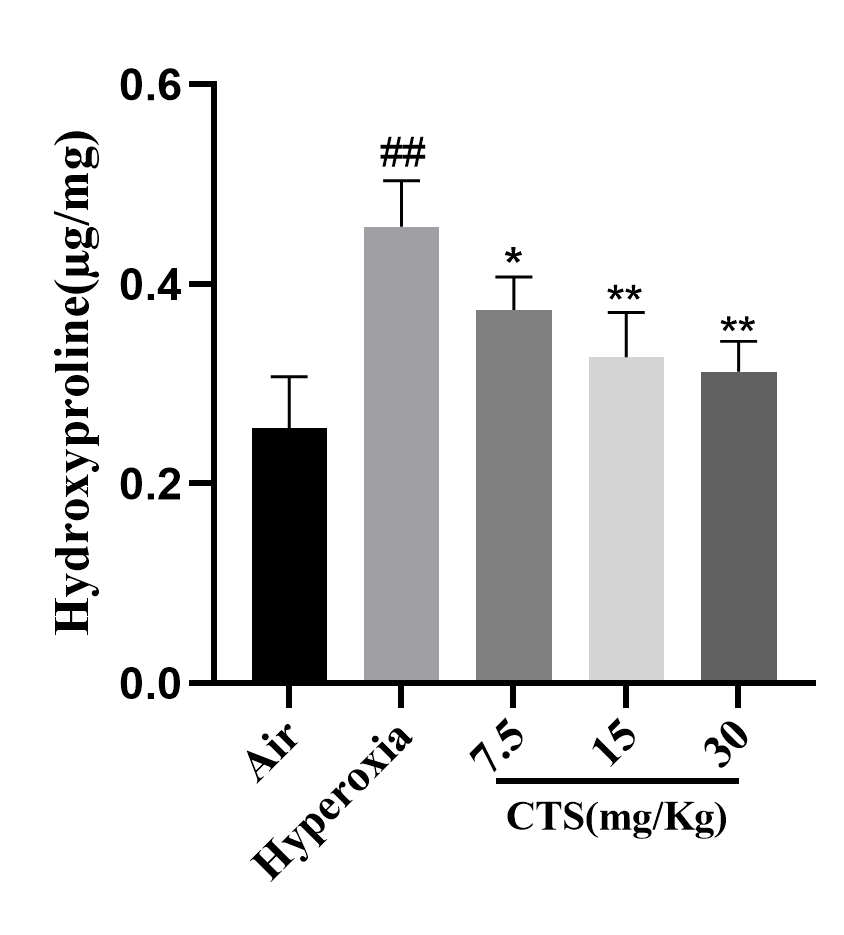

Supplement: Supplementary file 2 [file DataSheet1.ZIP › figures/HYP/HYP.tif]

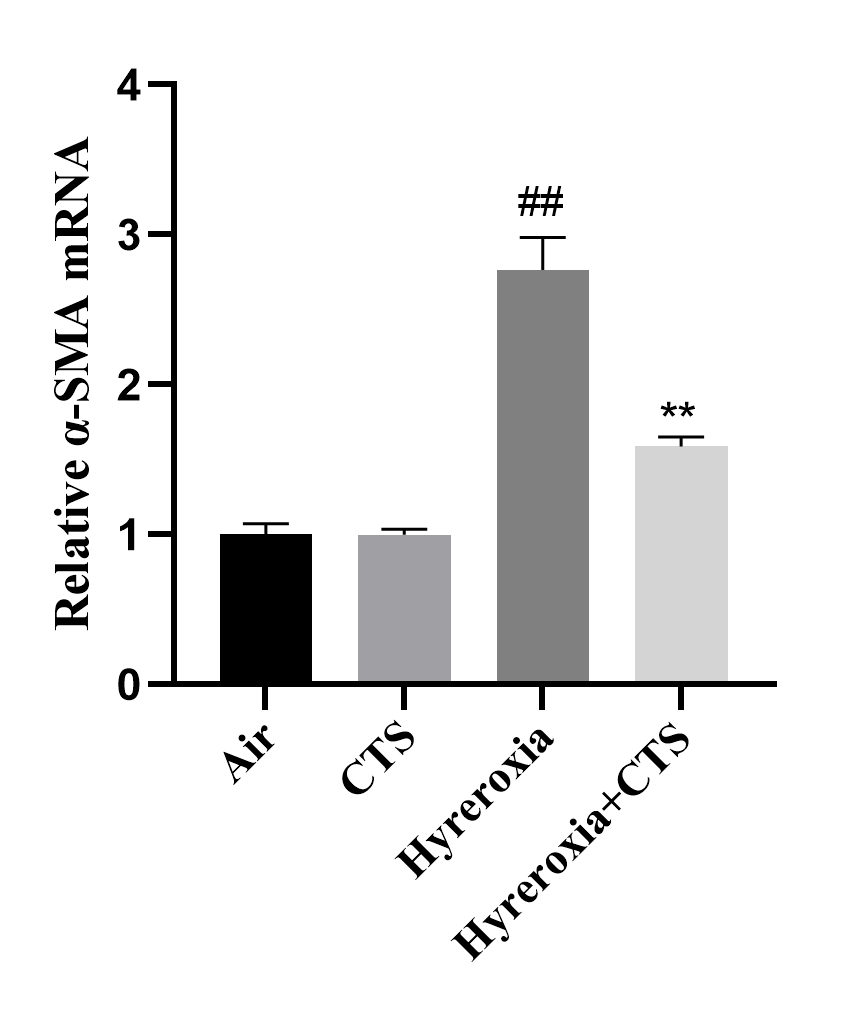

Supplement: Supplementary file 2 [file DataSheet1.ZIP › figures/Rt-qPCR/ACTA2 HFL-1.tif]

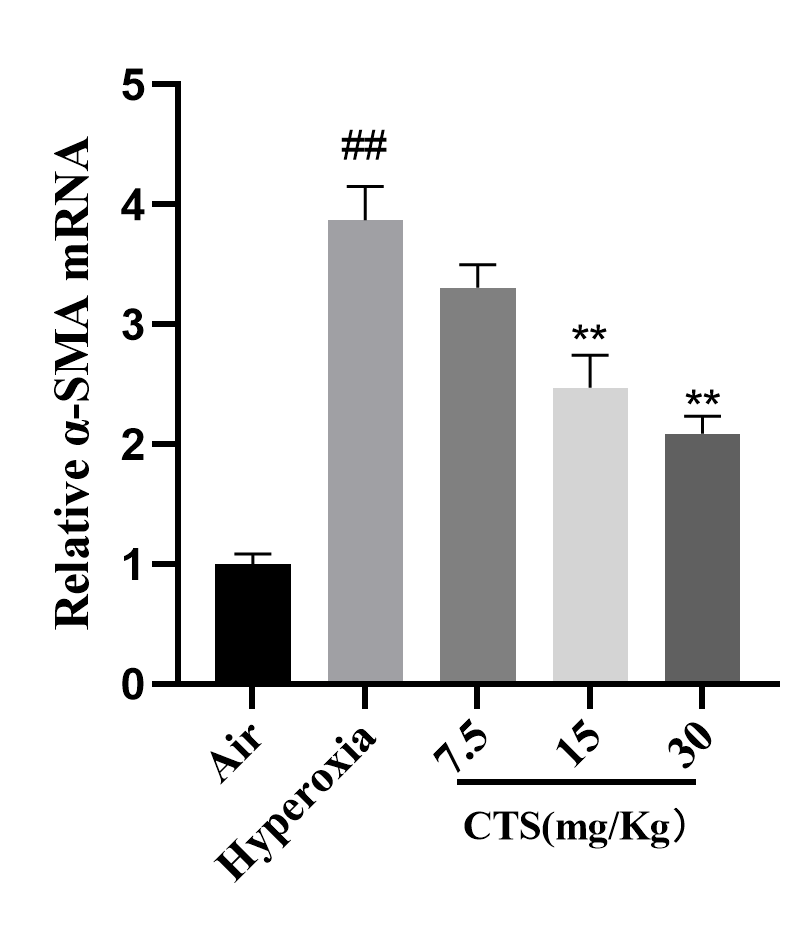

Supplement: Supplementary file 2 [file DataSheet1.ZIP › figures/Rt-qPCR/ACTA2.tif]

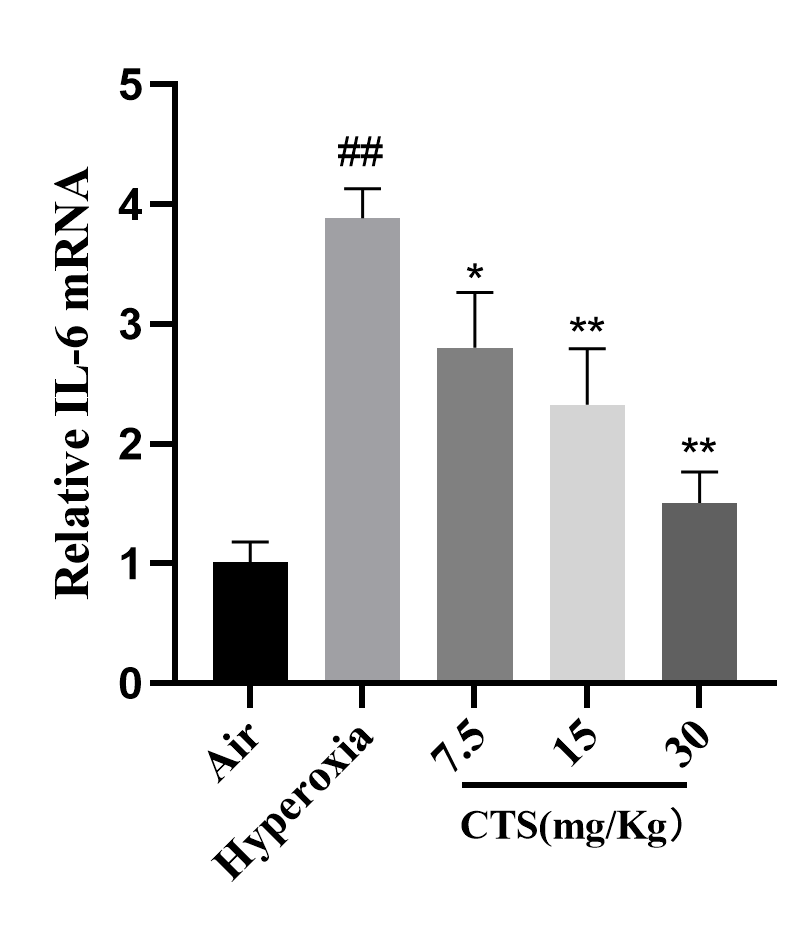

Supplement: Supplementary file 2 [file DataSheet1.ZIP › figures/Rt-qPCR/IL6.tif]

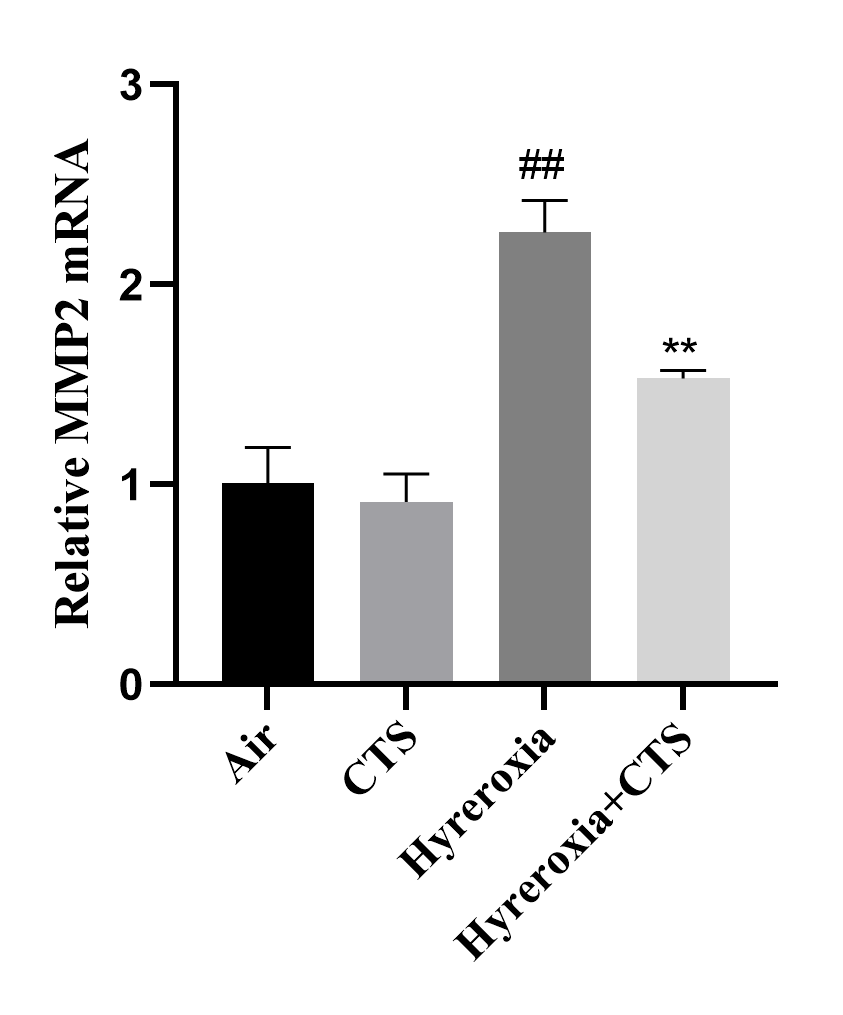

Supplement: Supplementary file 2 [file DataSheet1.ZIP › figures/Rt-qPCR/MMP2.tif]

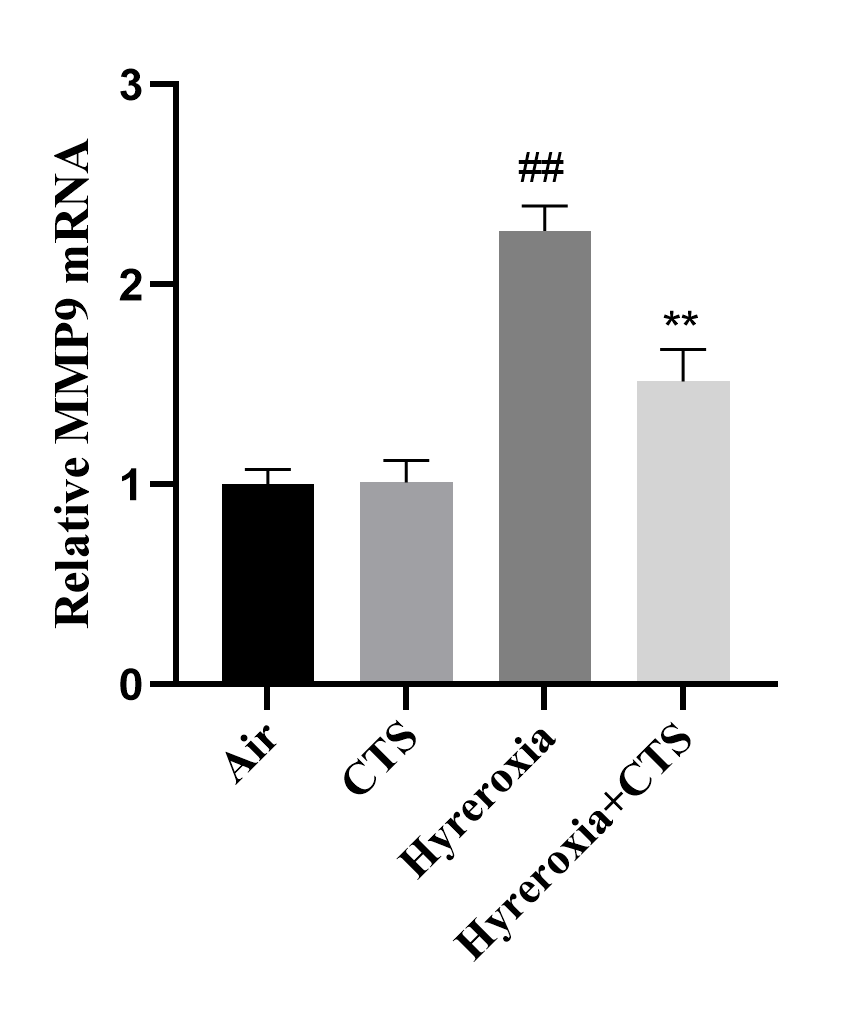

Supplement: Supplementary file 2 [file DataSheet1.ZIP › figures/Rt-qPCR/MMP9.tif]

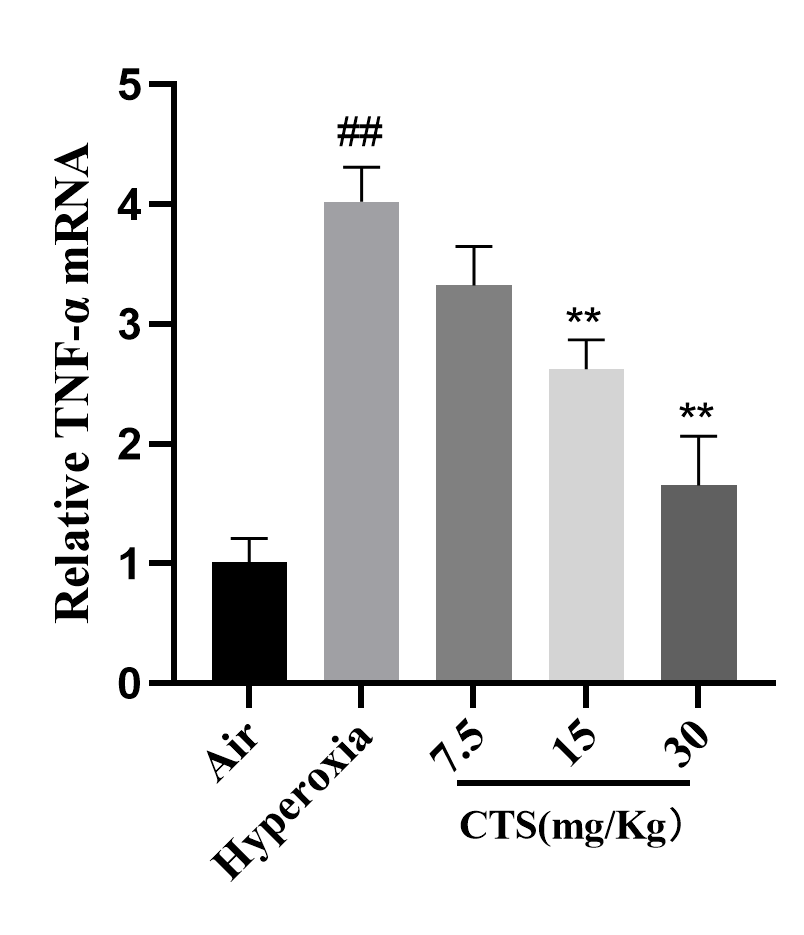

Supplement: Supplementary file 2 [file DataSheet1.ZIP › figures/Rt-qPCR/TNF-a┴.tif]

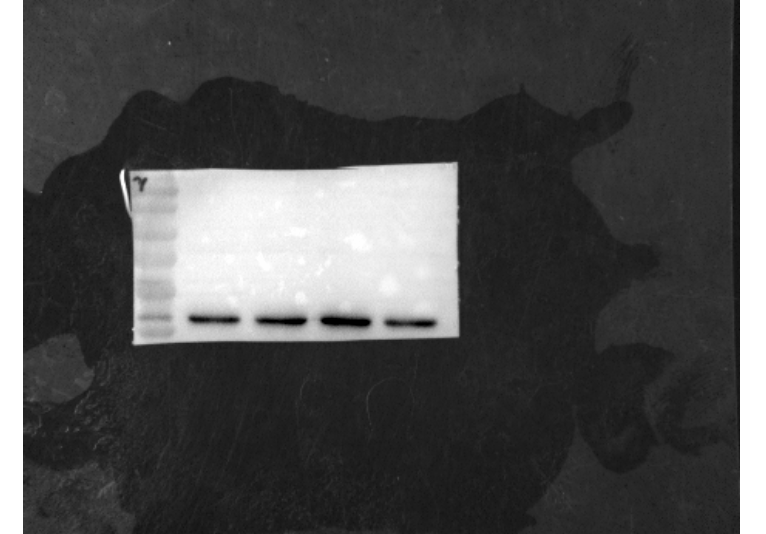

Supplement: Supplementary file 2 [file DataSheet1.ZIP › figures/WB/HFL-1 WB/ACTA2/1ACTA2.tif]

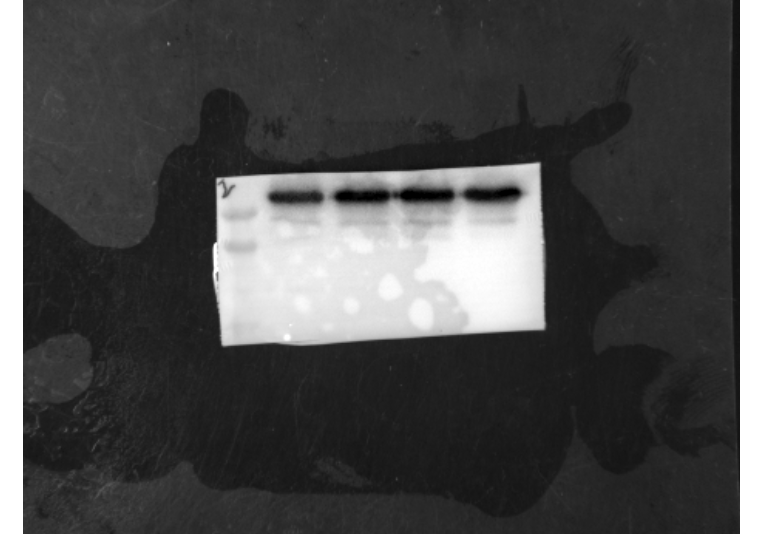

Supplement: Supplementary file 2 [file DataSheet1.ZIP › figures/WB/HFL-1 WB/ACTA2/1GAPDH.tif]

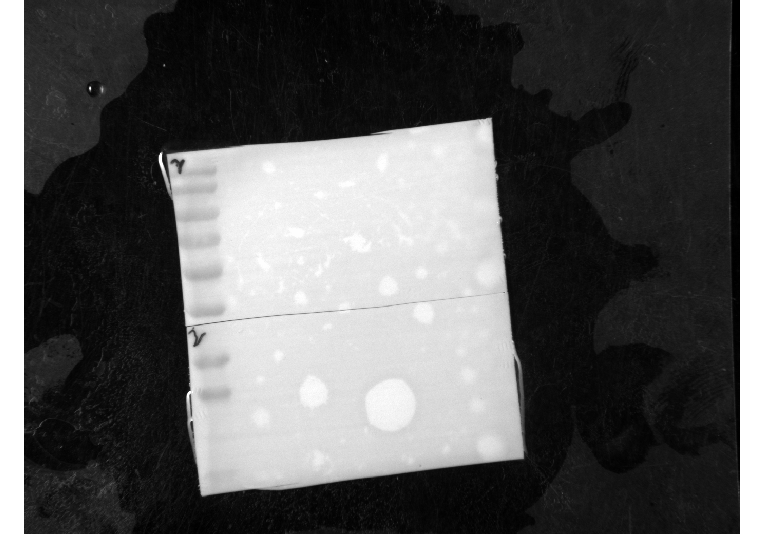

Supplement: Supplementary file 2 [file DataSheet1.ZIP › figures/WB/HFL-1 WB/ACTA2/1merge.tif]

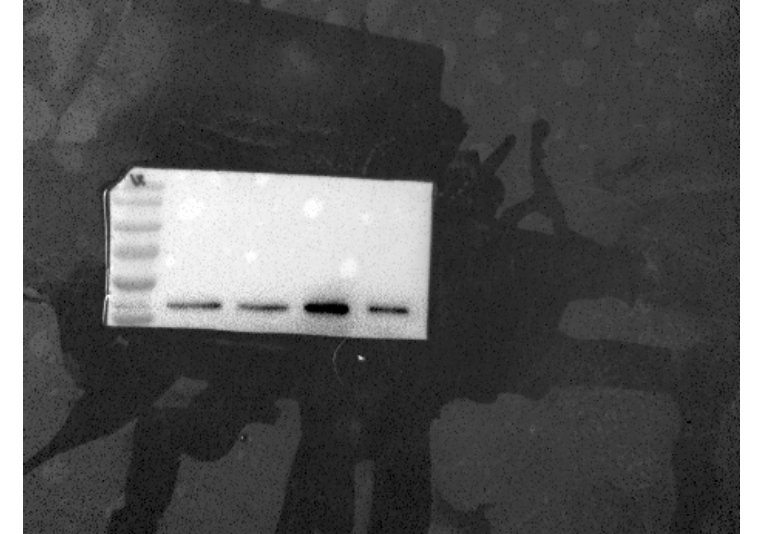

Supplement: Supplementary file 2 [file DataSheet1.ZIP › figures/WB/HFL-1 WB/ACTA2/2ACTA2.tif]

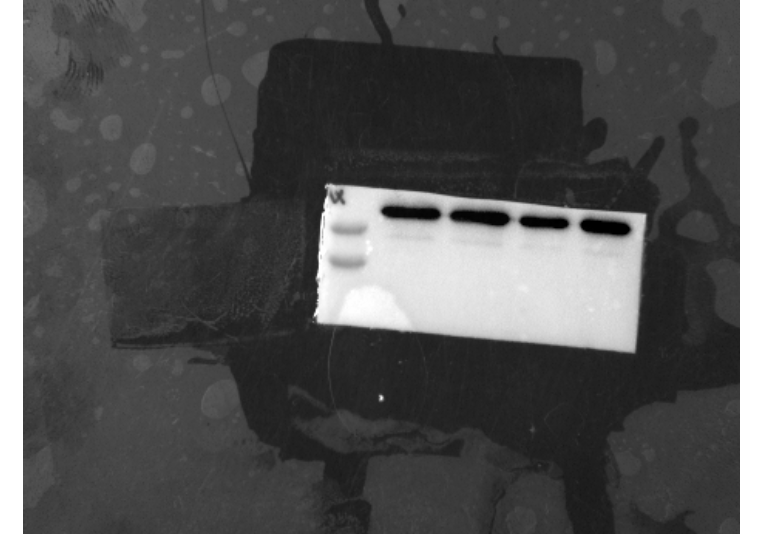

Supplement: Supplementary file 2 [file DataSheet1.ZIP › figures/WB/HFL-1 WB/ACTA2/2GAPDH.tif]

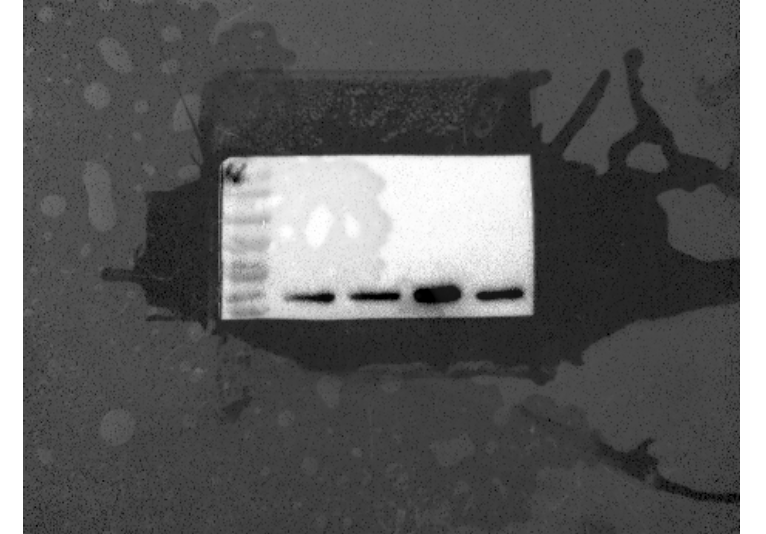

Supplement: Supplementary file 2 [file DataSheet1.ZIP › figures/WB/HFL-1 WB/ACTA2/3ACTA2.tif]

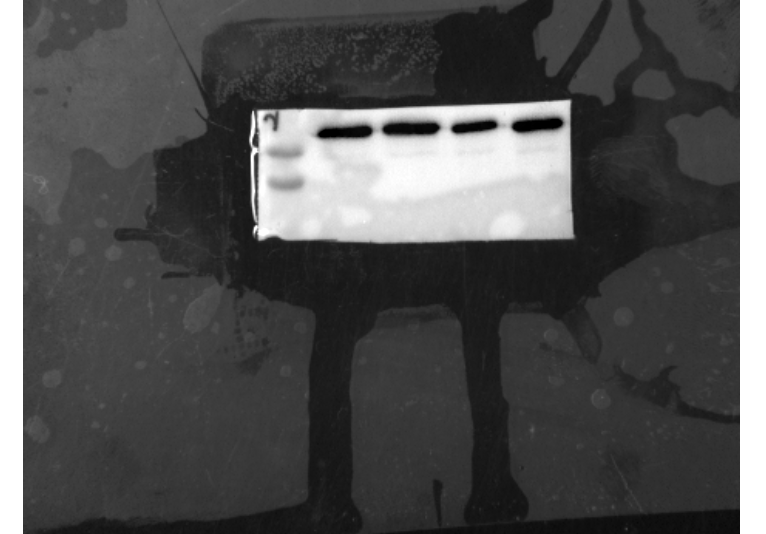

Supplement: Supplementary file 2 [file DataSheet1.ZIP › figures/WB/HFL-1 WB/ACTA2/3GAPDH.tif]

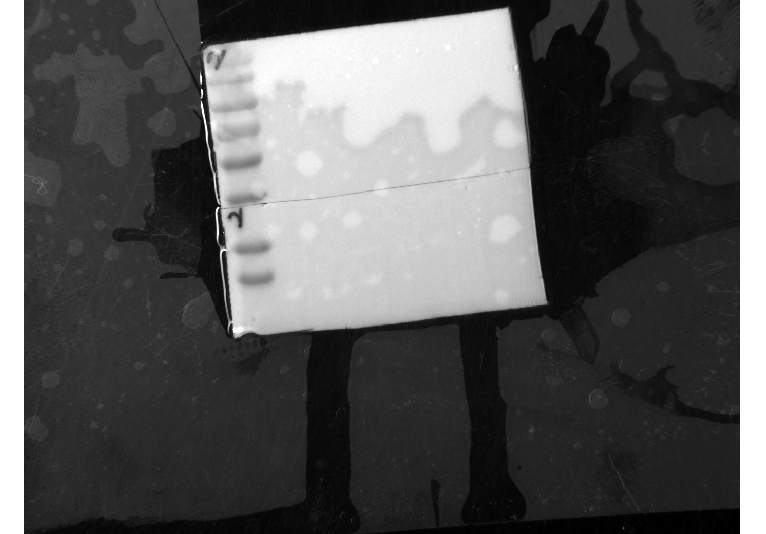

Supplement: Supplementary file 2 [file DataSheet1.ZIP › figures/WB/HFL-1 WB/ACTA2/3merge.tif]

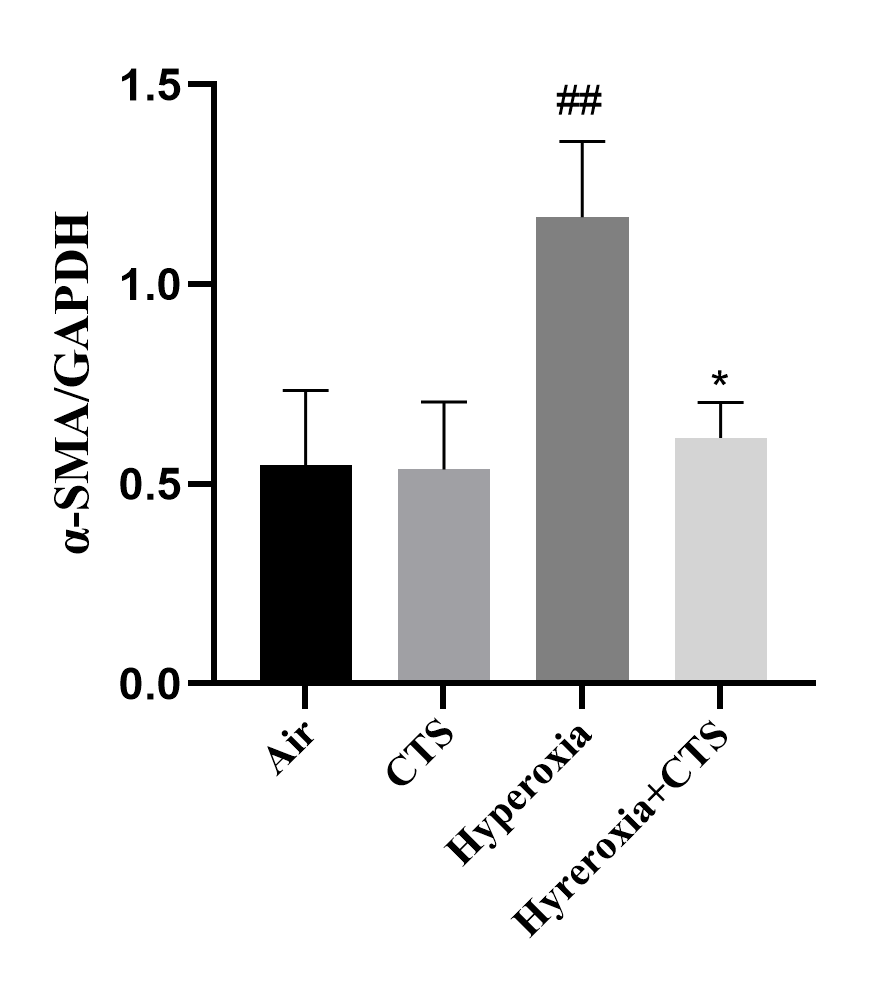

Supplement: Supplementary file 2 [file DataSheet1.ZIP › figures/WB/HFL-1 WB/ACTA2/ACTA2.tif]

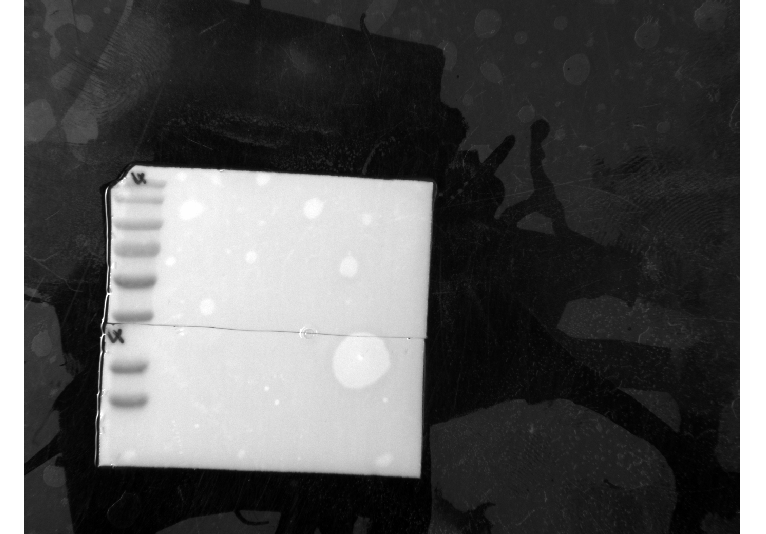

Supplement: Supplementary file 2 [file DataSheet1.ZIP › figures/WB/HFL-1 WB/ACTA2/merge2.tif]

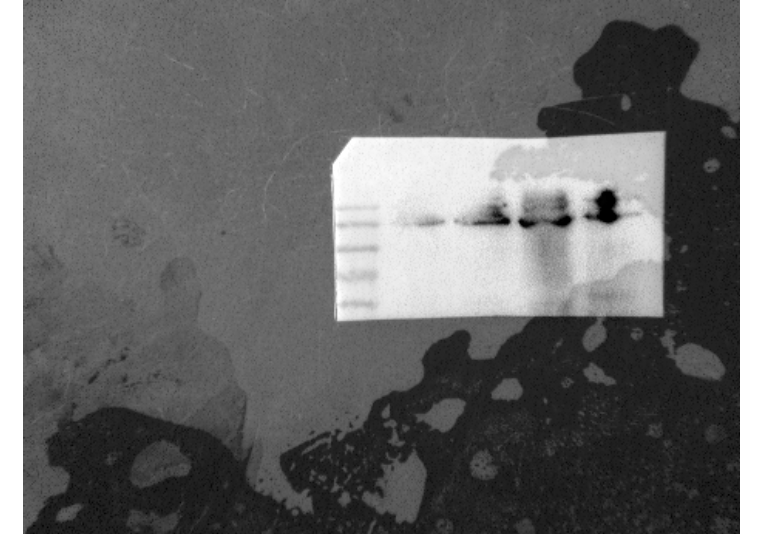

Supplement: Supplementary file 2 [file DataSheet1.ZIP › figures/WB/HFL-1 WB/COL-1/3col-1.tif]

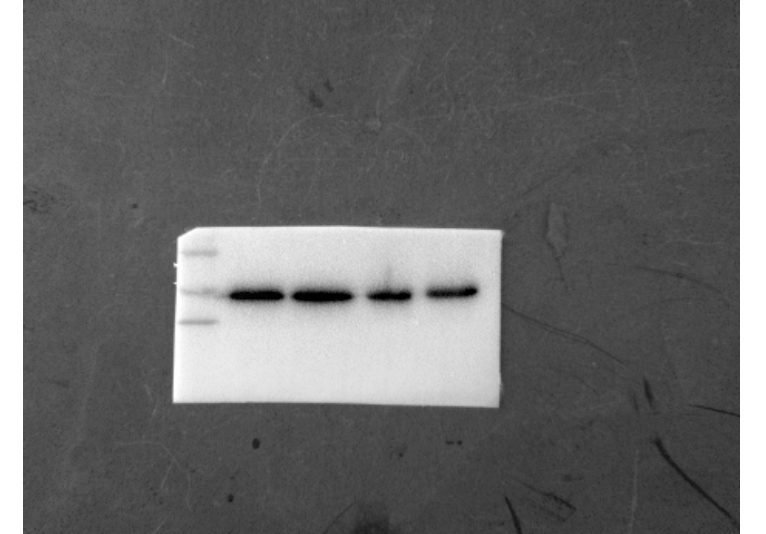

Supplement: Supplementary file 2 [file DataSheet1.ZIP › figures/WB/HFL-1 WB/COL-1/3GAPDH.tif]

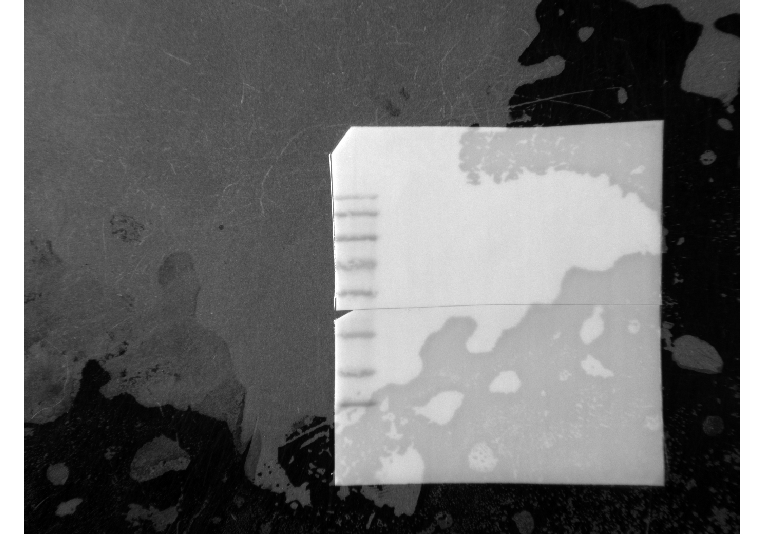

Supplement: Supplementary file 2 [file DataSheet1.ZIP › figures/WB/HFL-1 WB/COL-1/3merge.tif]

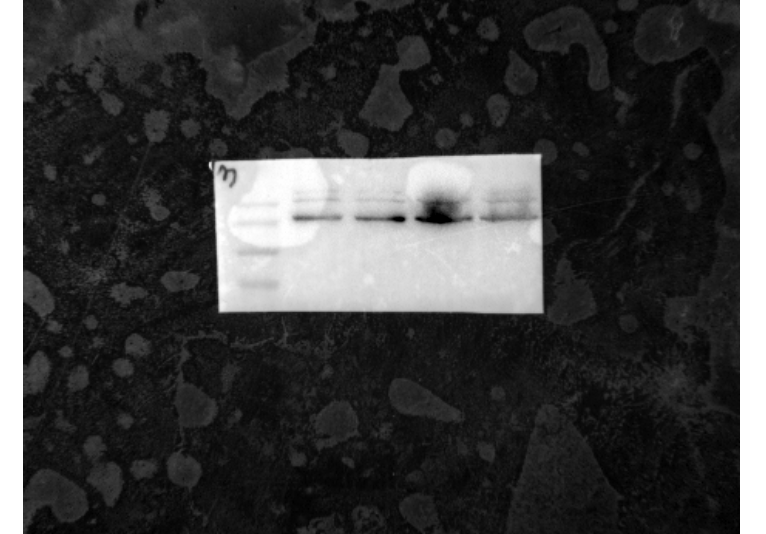

Supplement: Supplementary file 2 [file DataSheet1.ZIP › figures/WB/HFL-1 WB/COL-1/COL-1 1.tif]

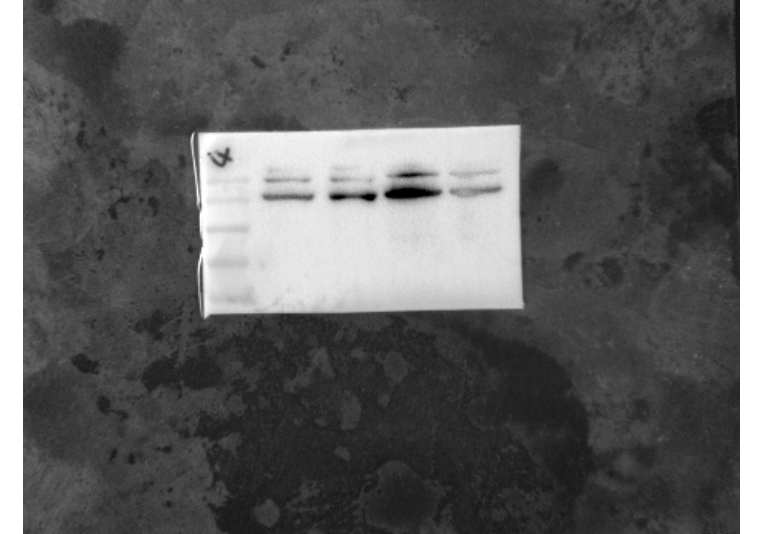

Supplement: Supplementary file 2 [file DataSheet1.ZIP › figures/WB/HFL-1 WB/COL-1/col-1 2.tif]

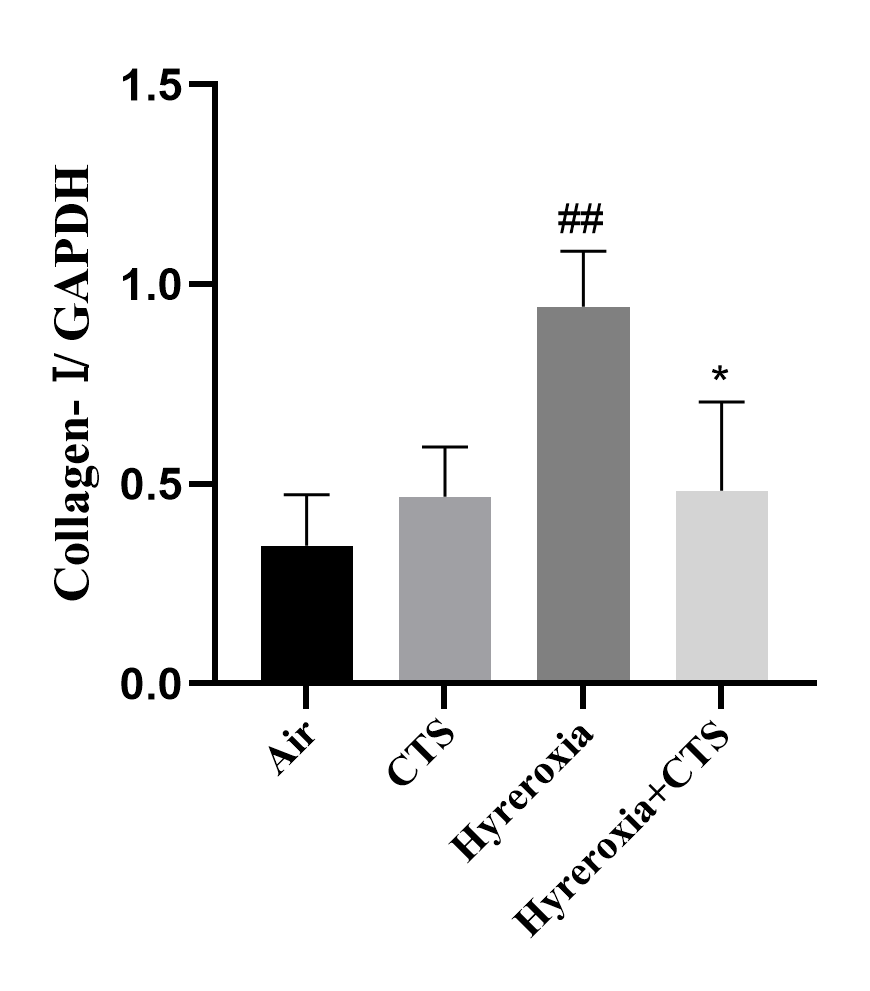

Supplement: Supplementary file 2 [file DataSheet1.ZIP › figures/WB/HFL-1 WB/COL-1/COL1.tif]

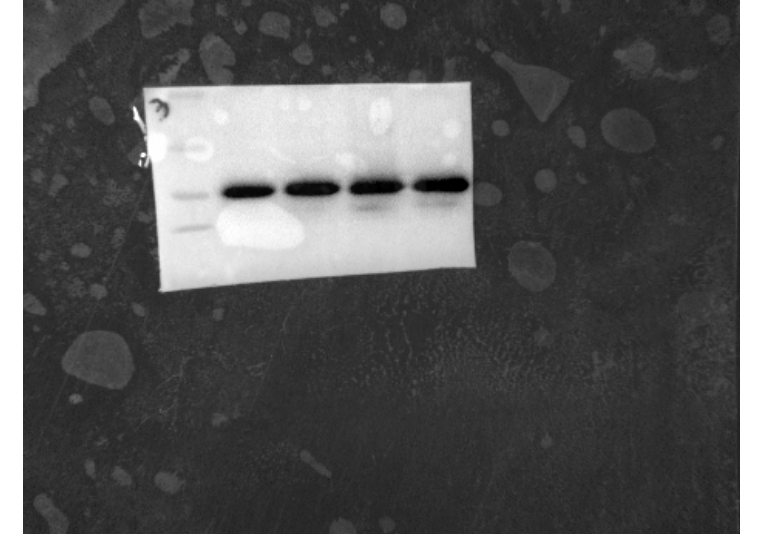

Supplement: Supplementary file 2 [file DataSheet1.ZIP › figures/WB/HFL-1 WB/COL-1/GAPDH1.tif]

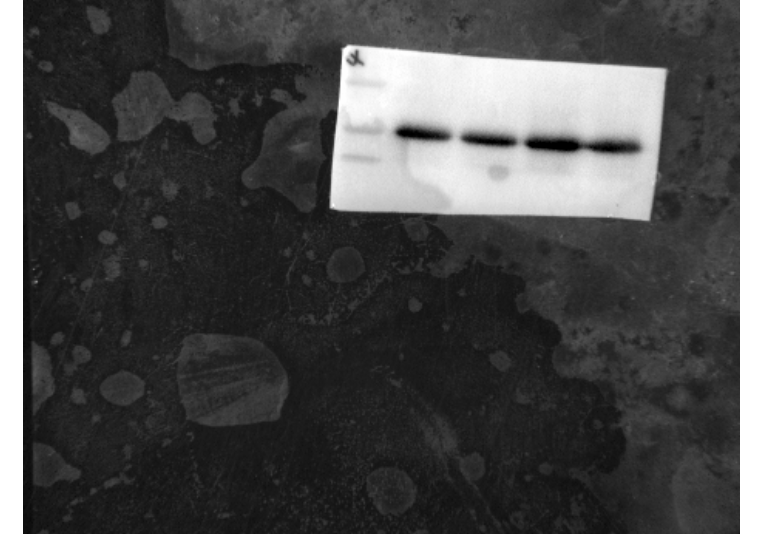

Supplement: Supplementary file 2 [file DataSheet1.ZIP › figures/WB/HFL-1 WB/COL-1/GAPDH2.tif]

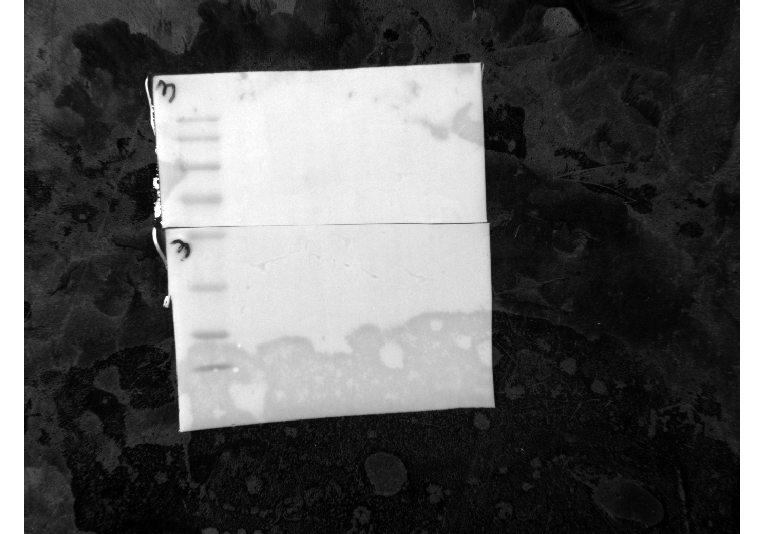

Supplement: Supplementary file 2 [file DataSheet1.ZIP › figures/WB/HFL-1 WB/COL-1/merge1.tif]

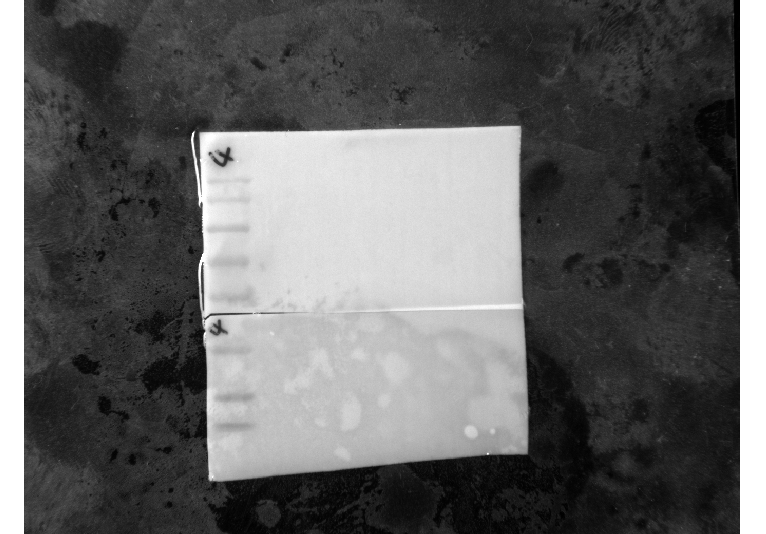

Supplement: Supplementary file 2 [file DataSheet1.ZIP › figures/WB/HFL-1 WB/COL-1/merge2.tif]

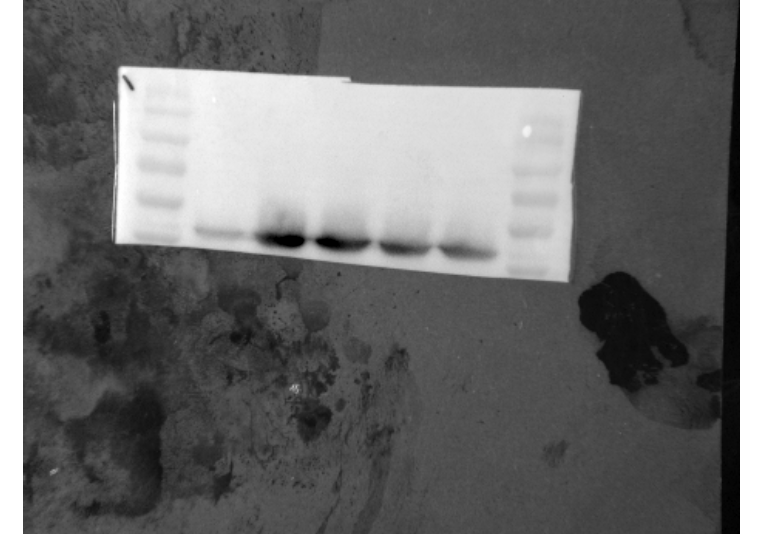

Supplement: Supplementary file 2 [file DataSheet1.ZIP › figures/WB/Rat WB/ACTA2/1/ACTA2.tif]

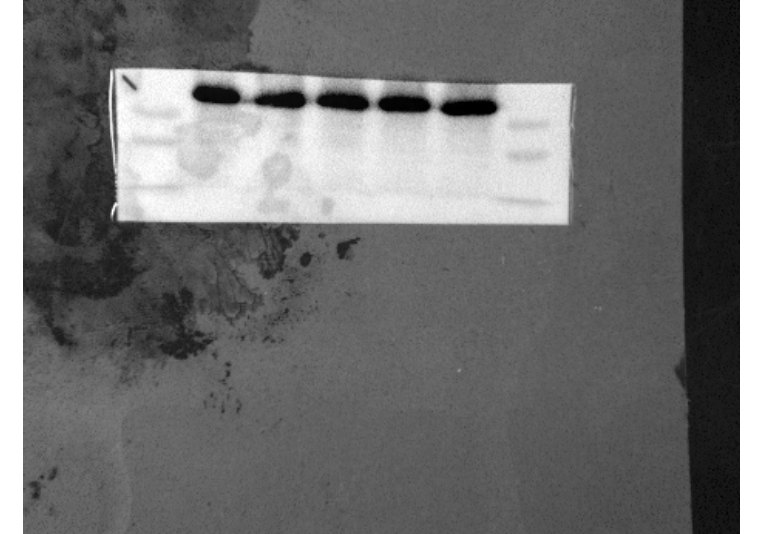

Supplement: Supplementary file 2 [file DataSheet1.ZIP › figures/WB/Rat WB/ACTA2/1/GAPDH.tif]

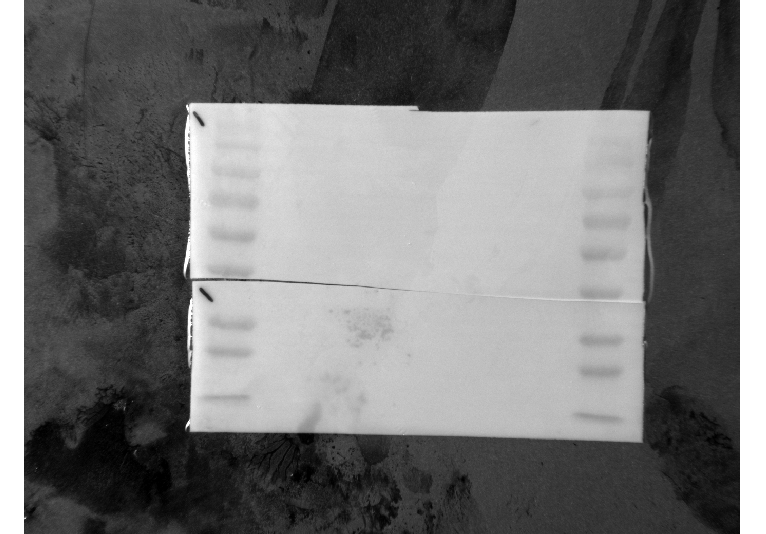

Supplement: Supplementary file 2 [file DataSheet1.ZIP › figures/WB/Rat WB/ACTA2/1/merge1.tif]

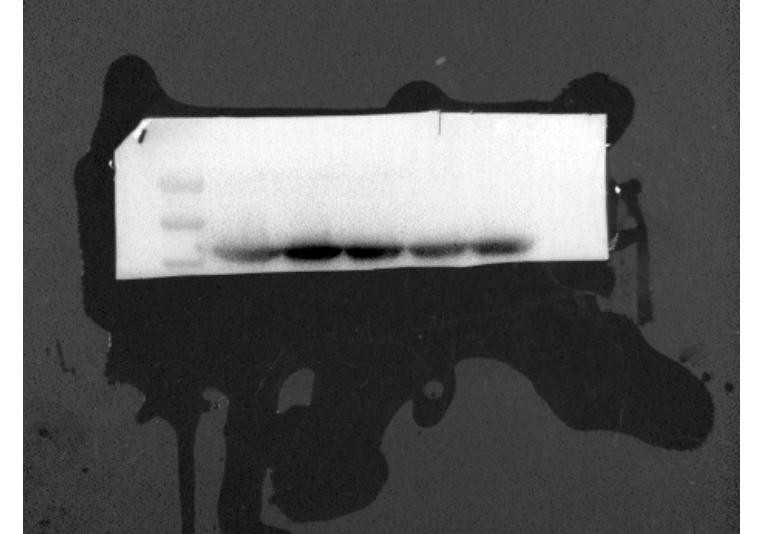

Supplement: Supplementary file 2 [file DataSheet1.ZIP › figures/WB/Rat WB/ACTA2/2/ACTA2.tif]

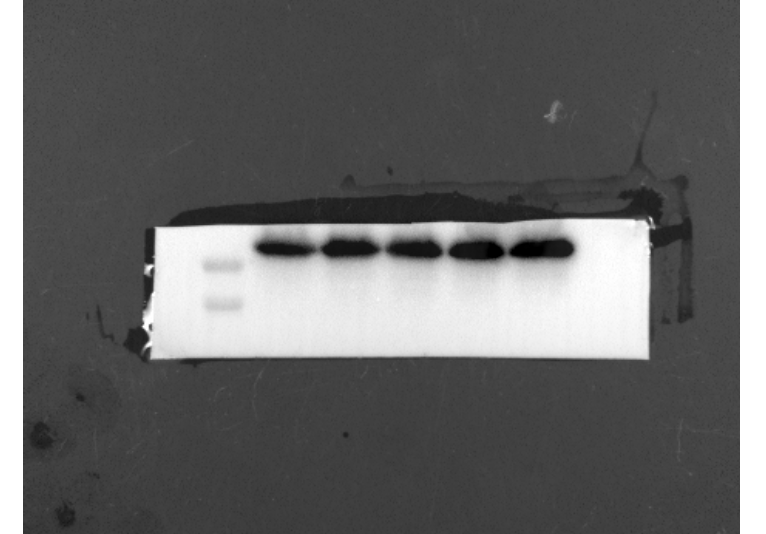

Supplement: Supplementary file 2 [file DataSheet1.ZIP › figures/WB/Rat WB/ACTA2/2/GAPDH.tif]

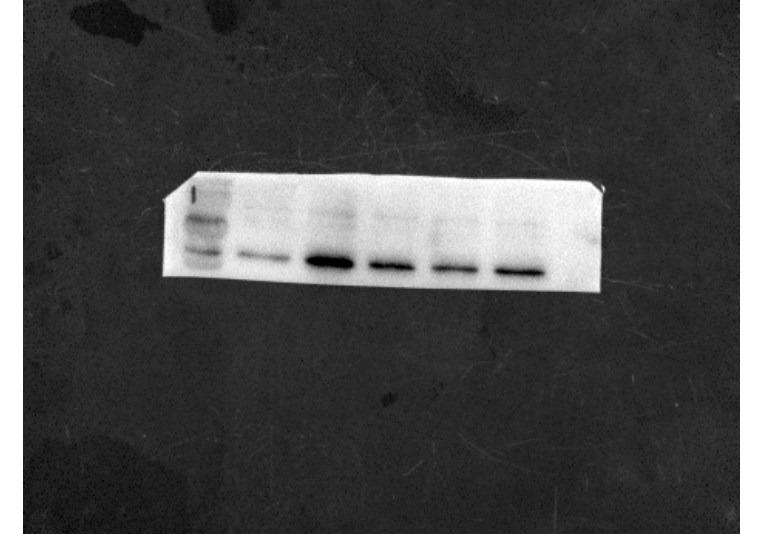

Supplement: Supplementary file 2 [file DataSheet1.ZIP › figures/WB/Rat WB/ACTA2/3/ACTA2.tif]

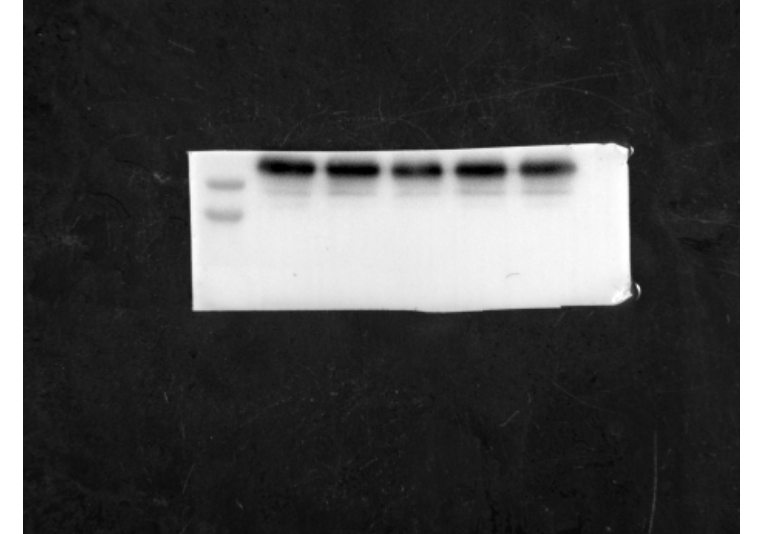

Supplement: Supplementary file 2 [file DataSheet1.ZIP › figures/WB/Rat WB/ACTA2/3/GAPDH.tif]

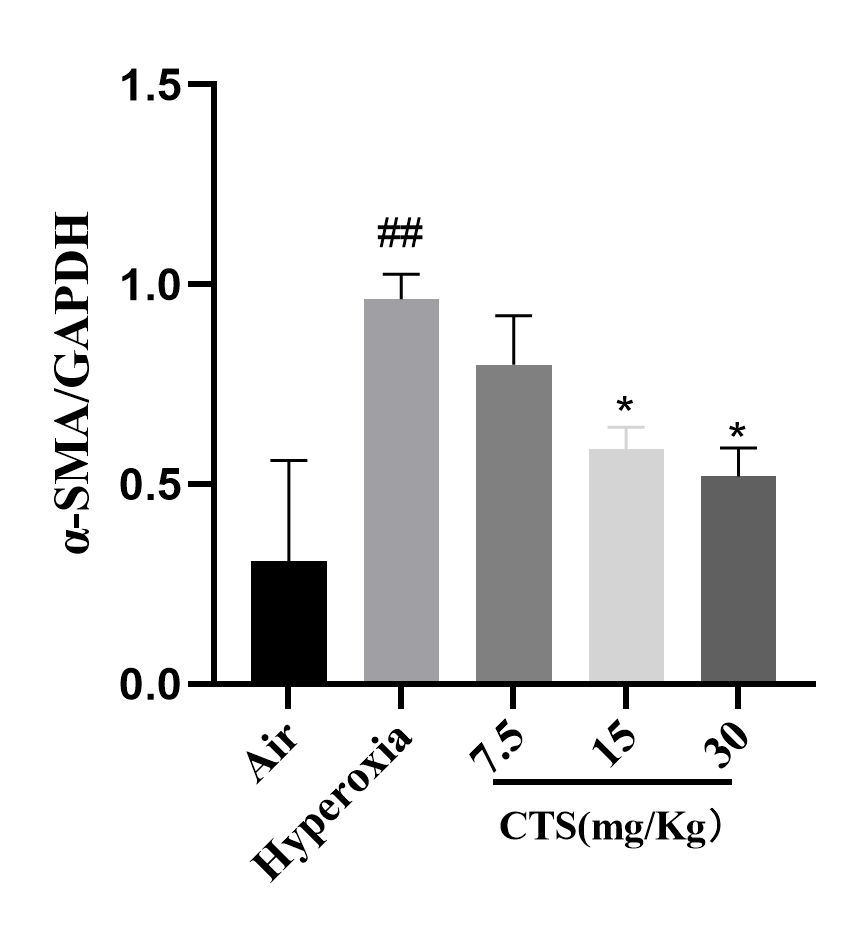

Supplement: Supplementary file 2 [file DataSheet1.ZIP › figures/WB/Rat WB/ACTA2/ACTA2╡░░╫.tif]

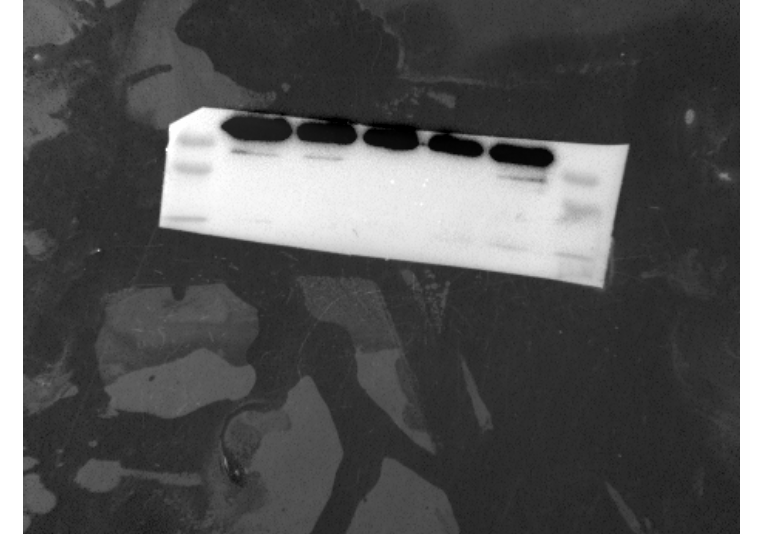

Supplement: Supplementary file 2 [file DataSheet1.ZIP › figures/WB/Rat WB/TGF-a┬1/GAPDH1.tif]

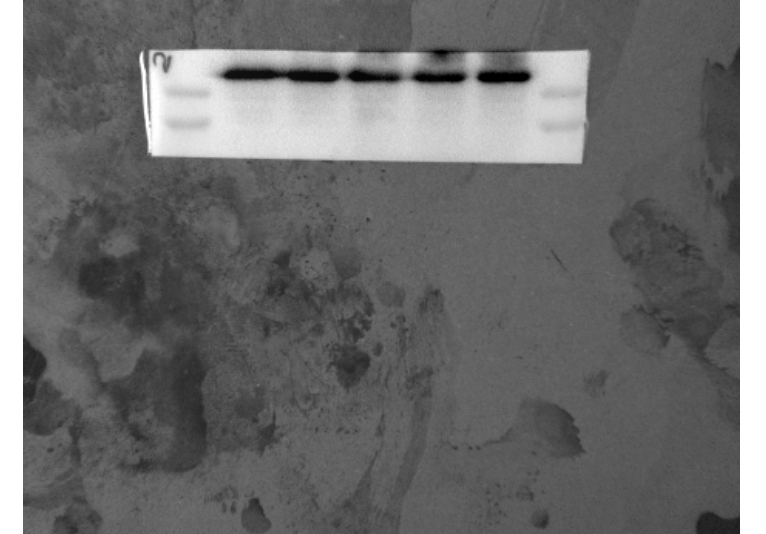

Supplement: Supplementary file 2 [file DataSheet1.ZIP › figures/WB/Rat WB/TGF-a┬1/GAPDH2.tif]

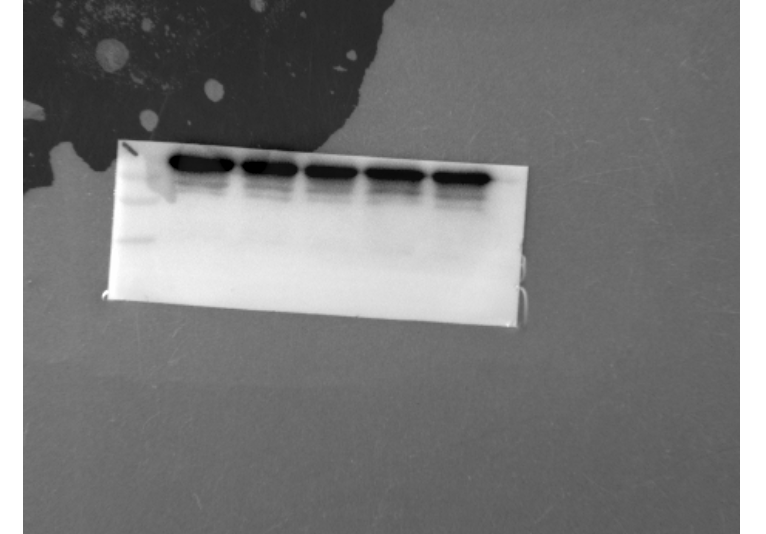

Supplement: Supplementary file 2 [file DataSheet1.ZIP › figures/WB/Rat WB/TGF-a┬1/GAPDH3.tif]

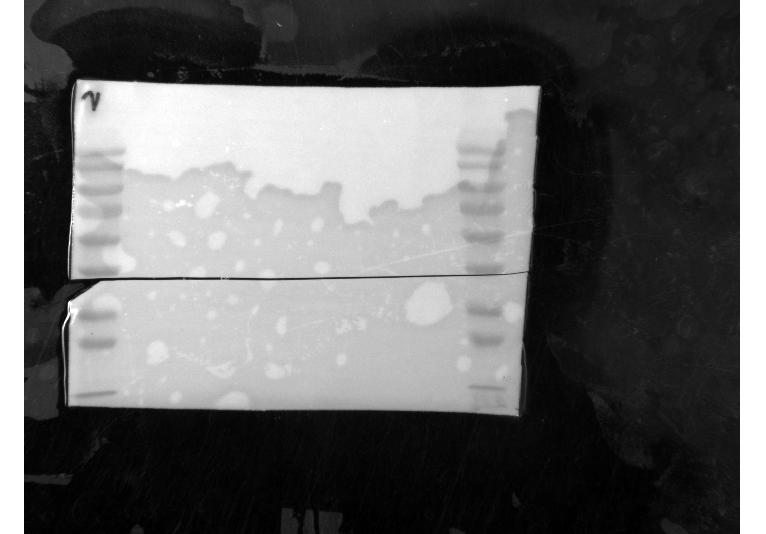

Supplement: Supplementary file 2 [file DataSheet1.ZIP › figures/WB/Rat WB/TGF-a┬1/merge1.tif]

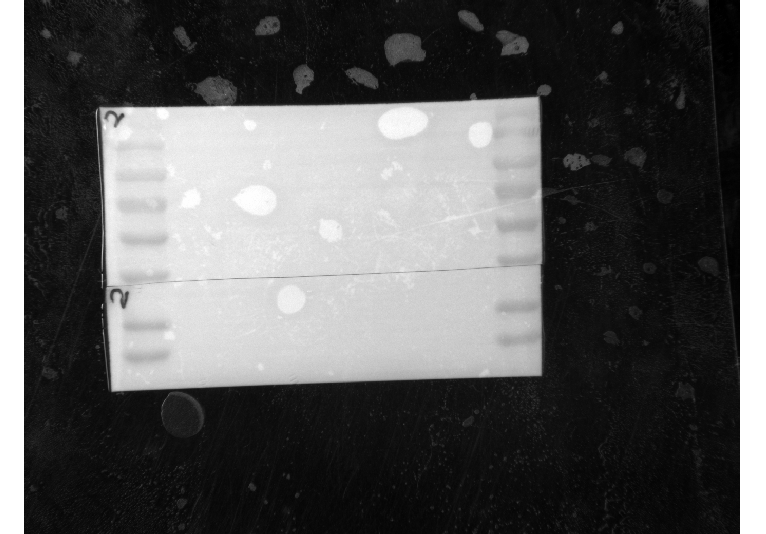

Supplement: Supplementary file 2 [file DataSheet1.ZIP › figures/WB/Rat WB/TGF-a┬1/merge2.tif]

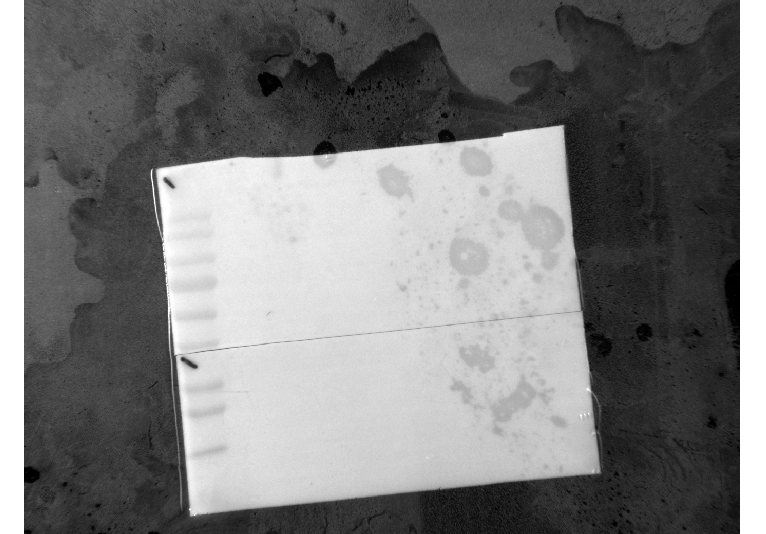

Supplement: Supplementary file 2 [file DataSheet1.ZIP › figures/WB/Rat WB/TGF-a┬1/merge3.tif]

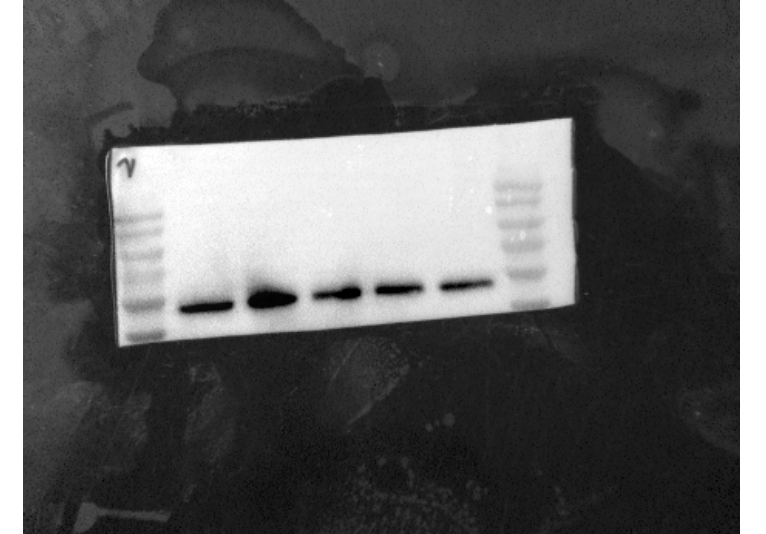

Supplement: Supplementary file 2 [file DataSheet1.ZIP › figures/WB/Rat WB/TGF-a┬1/TGFa┬1 1.tif]

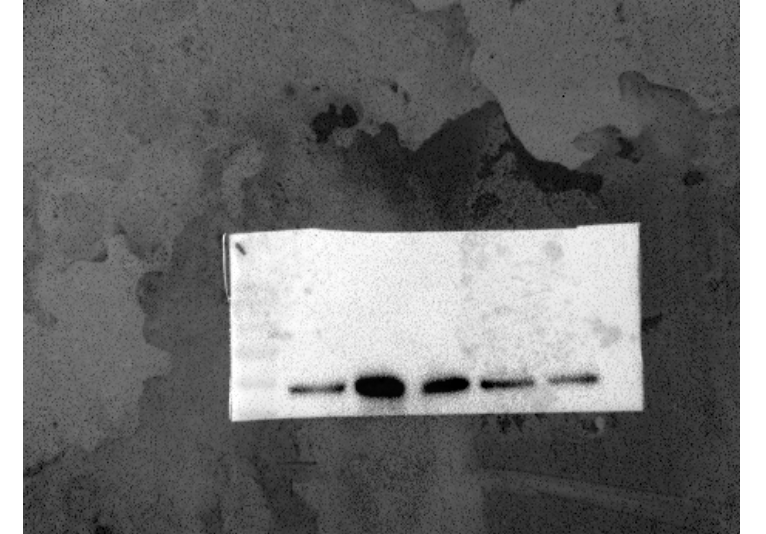

Supplement: Supplementary file 2 [file DataSheet1.ZIP › figures/WB/Rat WB/TGF-a┬1/TGFa┬1 3.tif]

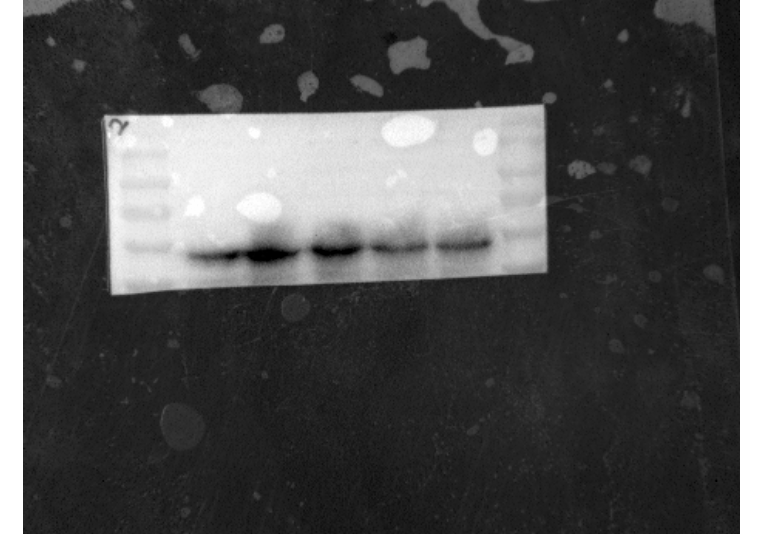

Supplement: Supplementary file 2 [file DataSheet1.ZIP › figures/WB/Rat WB/TGF-a┬1/TGFa┬12.tif]

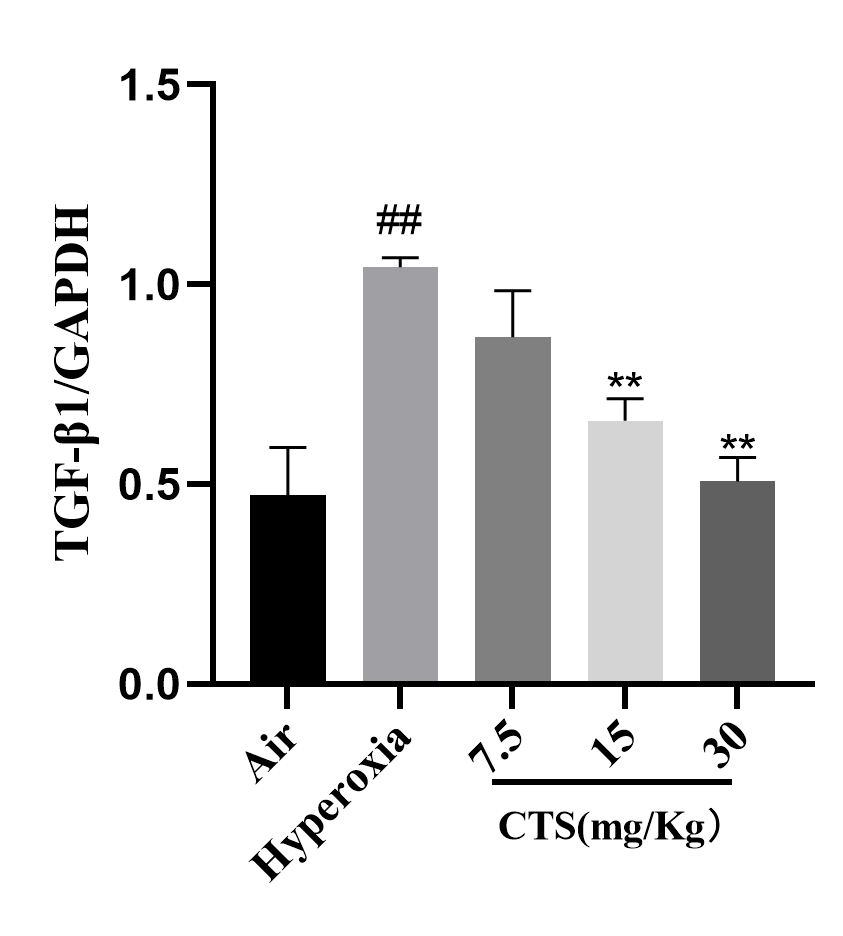

Supplement: Supplementary file 2 [file DataSheet1.ZIP › figures/WB/Rat WB/TGF-a┬1/TGF-a┬1╡░░╫.tif]

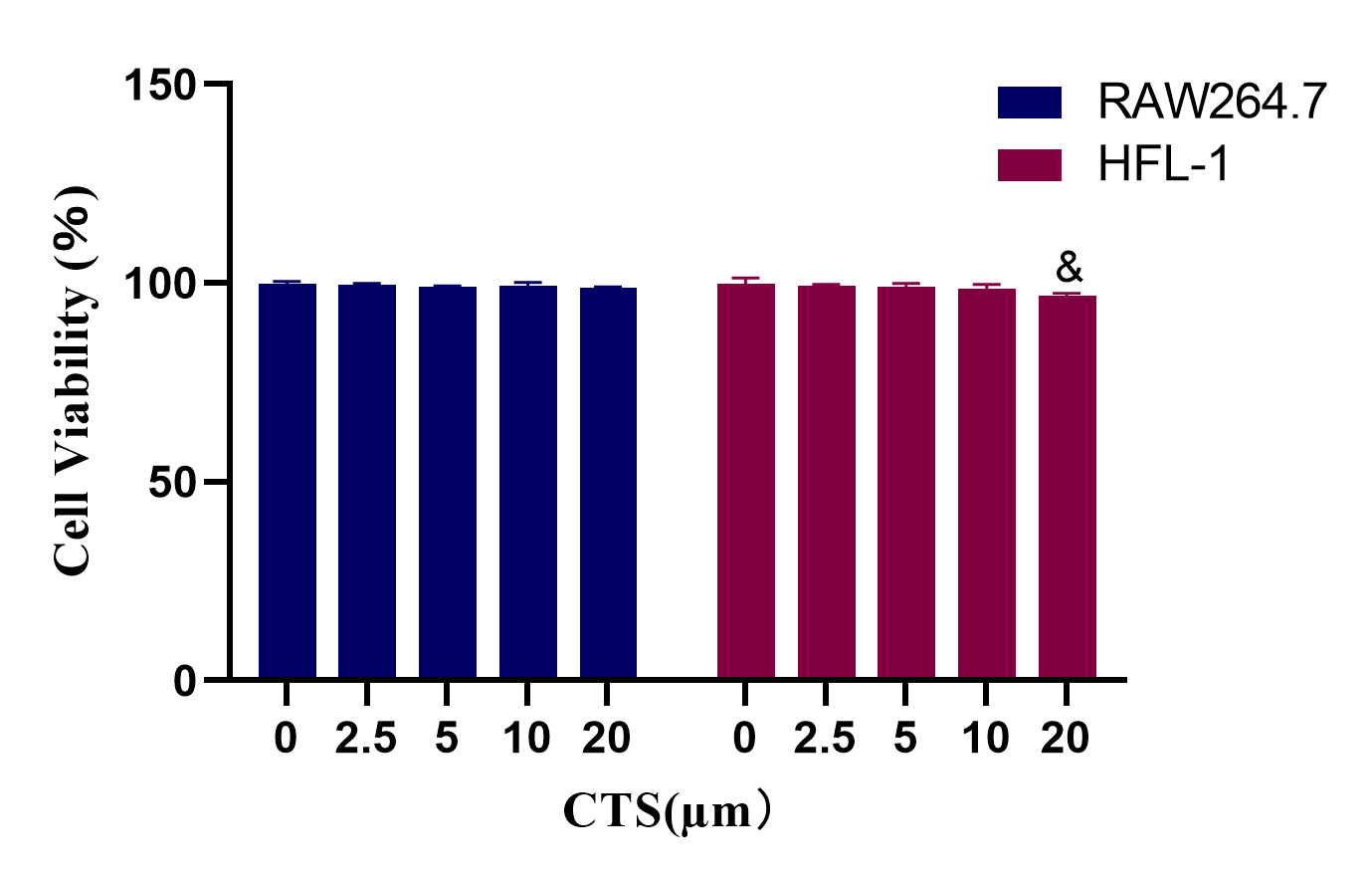

Supplement: Supplementary file 3 [file DataSheet2.ZIP › Data and figures/figures/CCK8/CCK8║╧▓ó.tif]

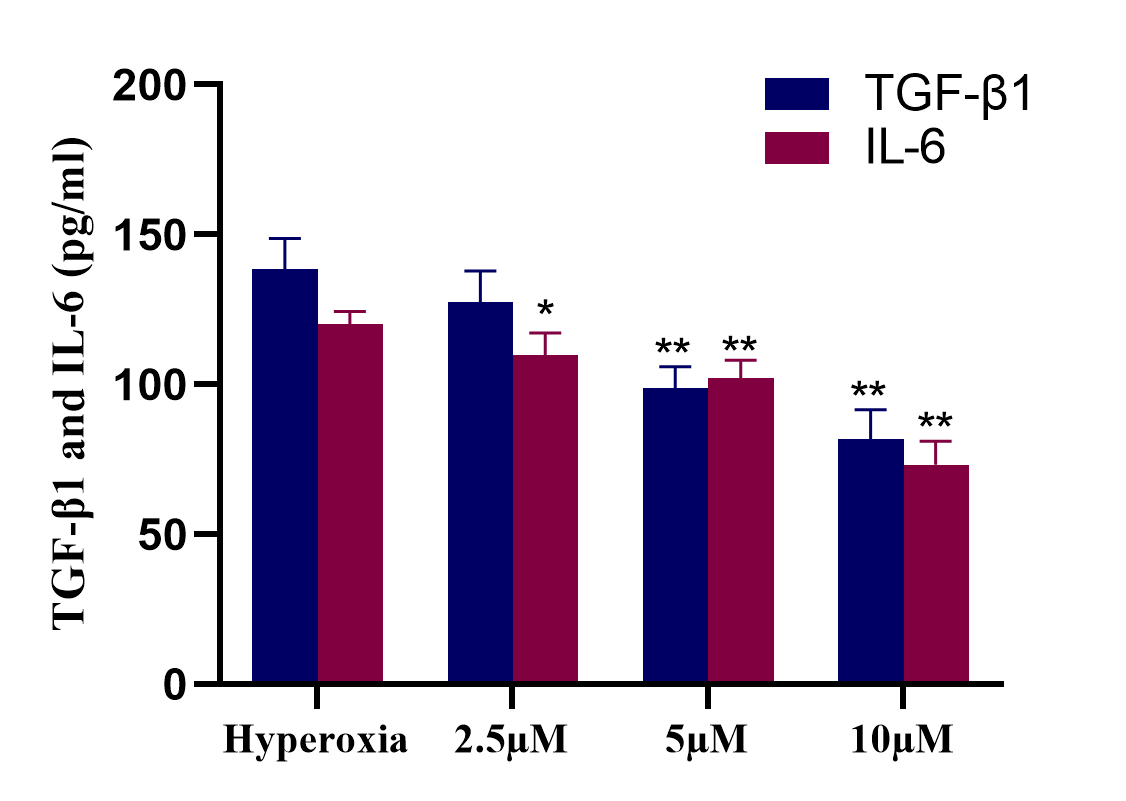

Supplement: Supplementary file 3 [file DataSheet2.ZIP › Data and figures/figures/ELISA/TGF and IL-6.tif]

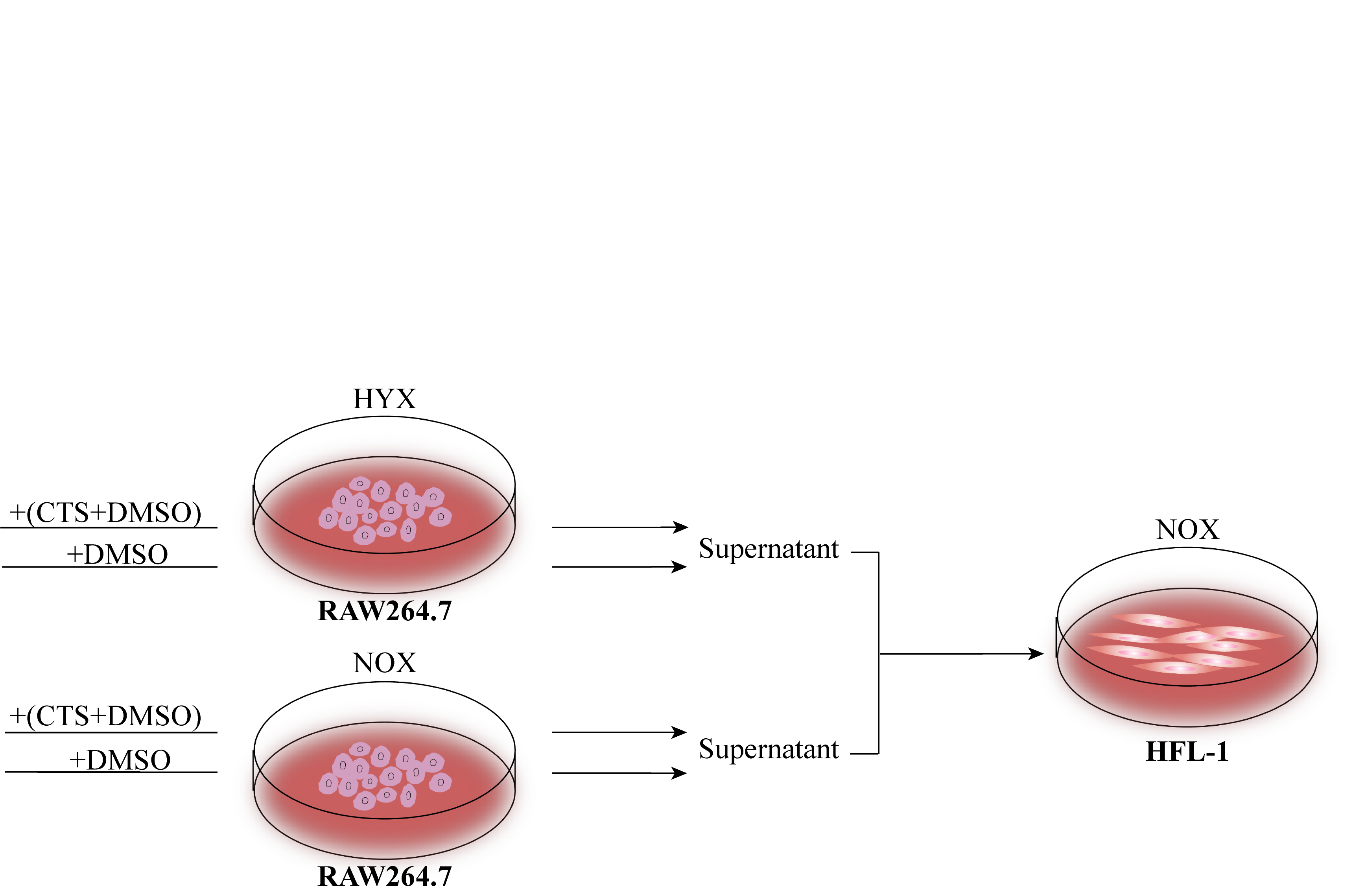

Supplement: Supplementary file 3 [file DataSheet2.ZIP › Data and figures/figures/Molding drawing/╠σ═Γ─ú╨══╝.tif]

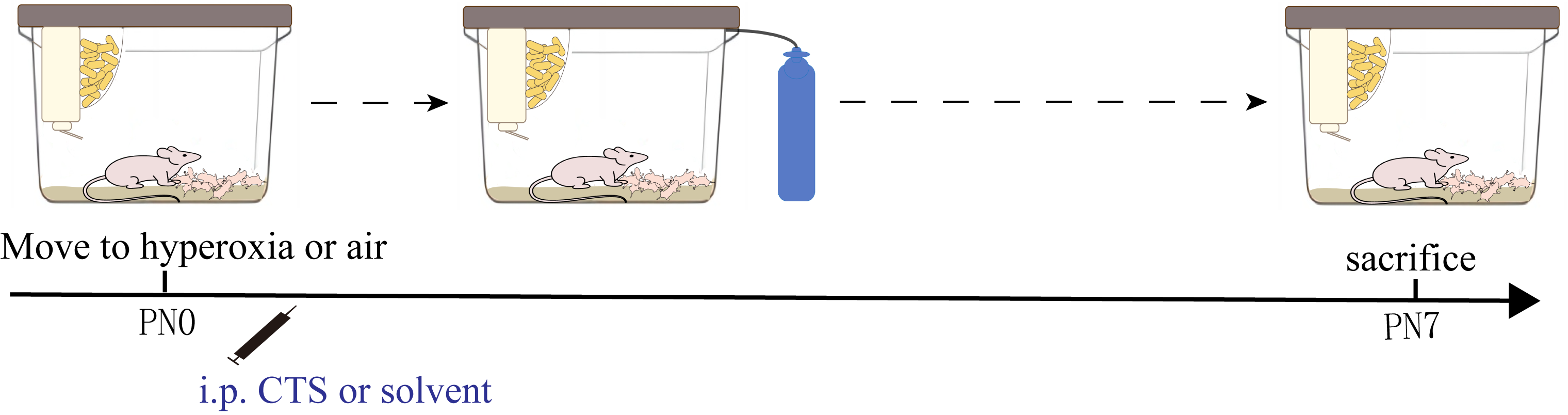

Supplement: Supplementary file 3 [file DataSheet2.ZIP › Data and figures/figures/Molding drawing/╘∞─ú╨▐╕─═╝2.tif]

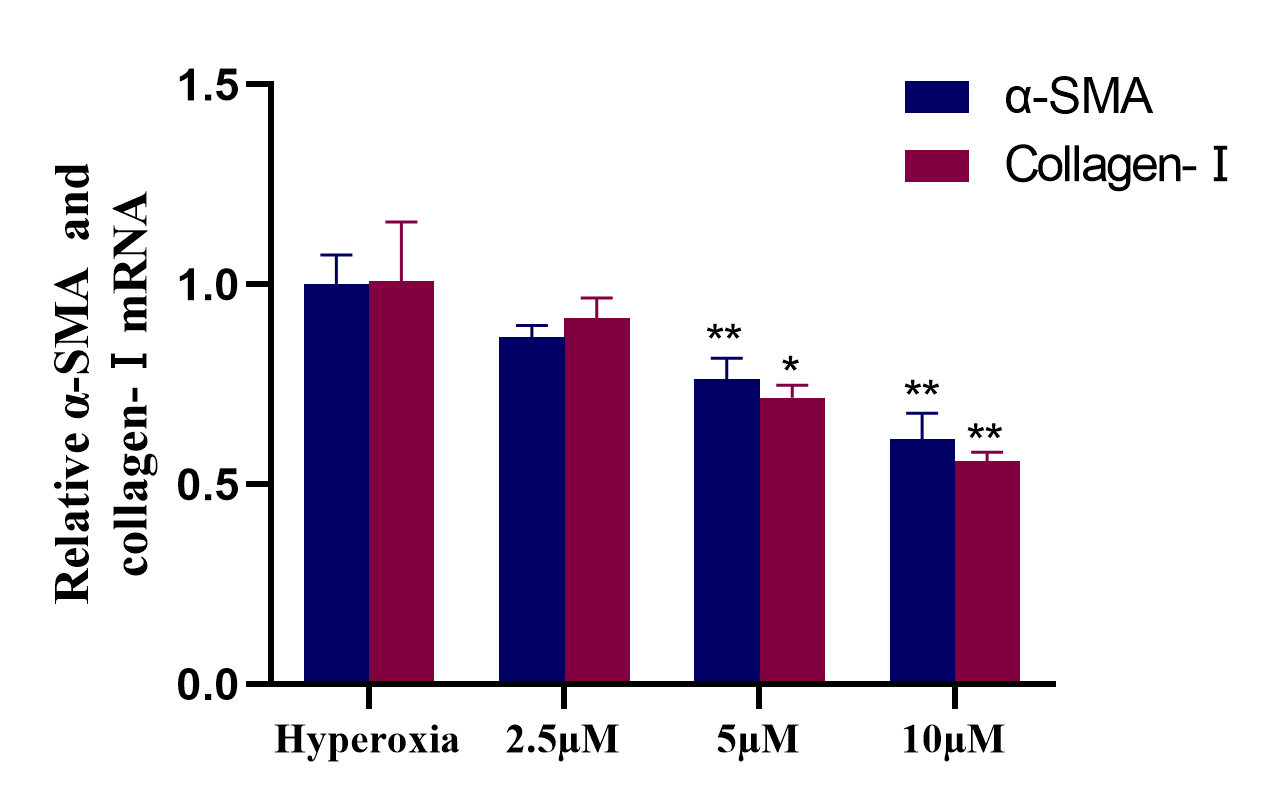

Supplement: Supplementary file 3 [file DataSheet2.ZIP › Data and figures/figures/PCR/ACTA2and COL1.tif]

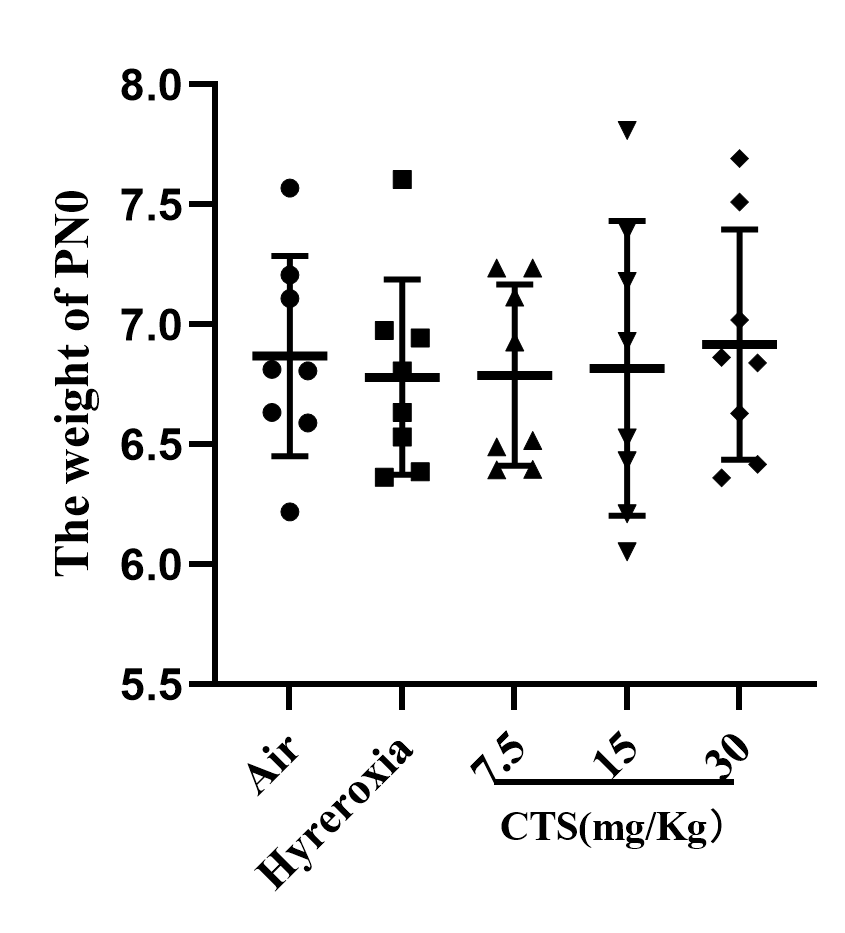

Supplement: Supplementary file 3 [file DataSheet2.ZIP › Data and figures/figures/weight/PN0╠σ╓╪.tif]

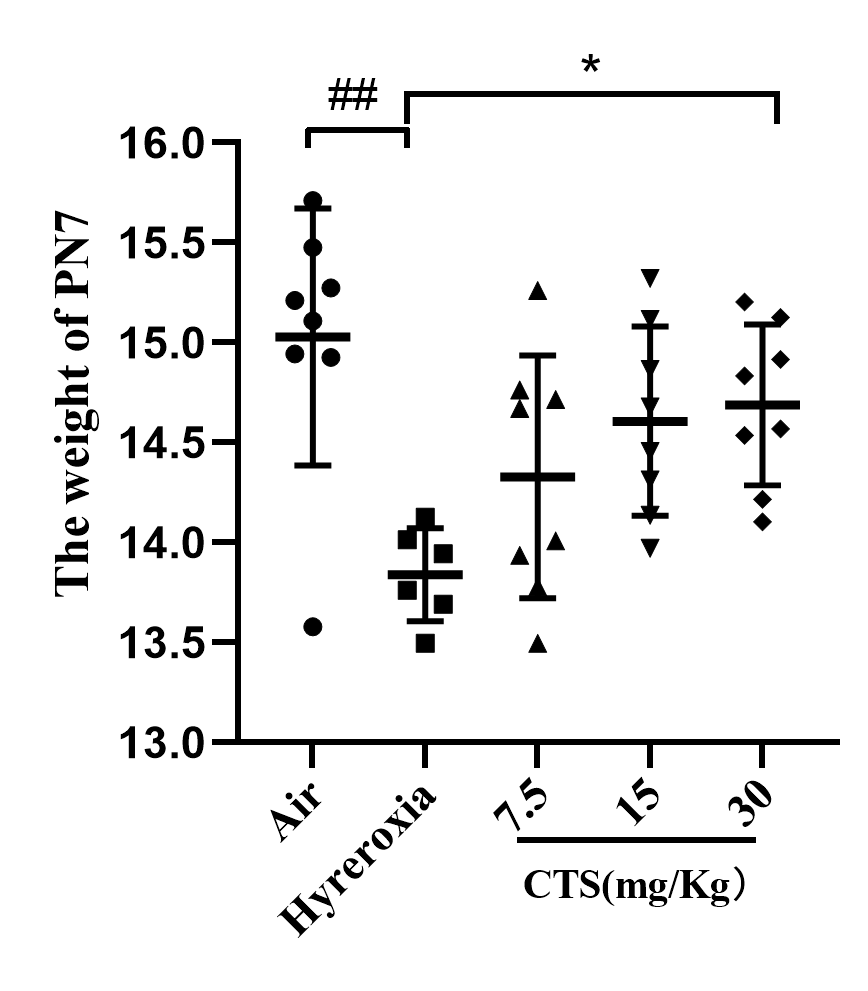

Supplement: Supplementary file 3 [file DataSheet2.ZIP › Data and figures/figures/weight/PN7╠σ╓╪.tif]
